# Follicular mediated etodolac phosalosomal gel for contact dermatitis alleviation, insights from optimization to *in-vivo* appraisal

**DOI:** 10.1038/s41598-024-71456-6

**Published:** 2024-09-18

**Authors:** Noha Khalifa Abo Aasy, Doaa Ragab, Marwa Ahmed Sallam, Kadria A. Elkhodairy

**Affiliations:** Department of Industrial Pharmacy, Faculty of Pharmacy, Alexandria University, 1 Khartoum Square, Azarita, Post Office, P.O. Box 21521, Alexandria, Egypt

**Keywords:** Hyaluronic acid, Contact dermatitis, Phosalosomes, Nociception, Anti-inflammatory, Skin irritation, Diseases, Health occupations, Rheumatology, Nanoscience and technology

## Abstract

Despite its long history as a preferential cyclooxygenase-2 inhibitor, the topical application of etodolac in inflammatory disorders does not achieve the desired clinical efficiency because of its poor water solubility and poor skin permeation. In the ongoing study, phosalosomes were designed to mitigate the etodolac drawbacks and to enhance its skin localization. Hyaluronic acid was utilized to prepare a dermal gel for the alleviation of skin inflammation. Etodolac loaded hyaluronic acid phosalosomal gel had a sustainable release profile and 10.59-fold enhanced skin retention compared to free etodolac, with boosted skin tolerability on histopathological examination after acute and chronic applications. Confocal laser microscopy imaging indicated that the etodolac amounts accumulated in the liver and kidney following dermal application were 29 and 5.7-fold lower than those following the systemic dose, respectively. For in vivo studies, etodolac loaded hyaluronic acid phosalosomal gel presented superior anti-oedemic and significant anti-nociception potential. The promising homogenous localization highlighted its potential for the delivery of lipophilic drugs for the targeted treatment of other localized skin disorders.

## Introduction

The inflammatory response is one of the major pathogenic module in various diseases that affects the innate immune response. The production of the inflammatory mediators as inflammatory cytokines contributes to the upsurge of various chronic diseases such as rheumatoid arthritis, osteoarthritis, inflammatory bowel diseases, inflammatory skin diseases (atopic dermatitis, psoriasis) that oblige non-steroidal anti-inflammatory drugs (NSAIDs) or glucocorticoids as a cordial and continuous treatment; or acute diseases such as acute ischemic, airway inflammation, or trauma that defined by redness, swelling, pain and also mandates high doses of anti-inflammatory drugs^1–3^.

Contact dermatitis (CD) is considered as one of the acute or chronic cutaneous inflammatory disorder that attributable to the contact with an irritant (chemical or physical agents)^4^.The clinical manifestations of CD start shortly after the exposure to the irritant and feature as erythema, itching, pain, burning sensation, and may extend into tissue necrosis^5^. Due to that fact topical corticosteroids are the first line treatment of CD, and their known side effects as promotion of cell atrophy and reduction of tissue regeneration potential, the evolvement of safer treatment regimen should be considered^6^.

The skin played an increasingly important role as a non- invasive (surface area = 20 ft^2^) and effective route for both local and systemic drug administrations. Although skin provides its primary function in body protection from the surrounding environment, it also acts as a significant barrier to the penetration of exogenous molecules^7^. This is mainly because of the unique structure of the outermost stratum corneum layer, which is simply described as a series of layers of flattened corneocytes surrounded by a lipid envelope. One common pathway that is indicated to penetrate the stratum corneum, which could be assigned as an intercellular route, is through the lipids surrounding the corneocytes^8^; however, under certain conditions, the transcellular route through the corneocytes may be also applicable. Alternatively, the hair follicles, which transverse the stratum corneum, may be also a potential target for drug delivery through the skin layers. Interestingly, the penetration depth of different formulations across hair follicles is primarily dependent on the particle size. Nano-formulations with a diameter of 320 nm were observed to reside in hair follicles for 10 days (d); this indicate the ability of hair follicles to act as drug reservoirs for controlled release site-targeted dermal applications. For nanoparticles  < 200 nm, deep follicular deposition was confirmed followed by Langerhans cellular internalization; no internalization was observed for particles in the size range 750–1500 nm^9^.In the review of existing research works, several studies focus on the enhancement of follicular delivery of nanoparticles^7^. Lademann et al.^8^ investigated the effect of massage to promote the skin delivery of nanoparticles (300–600 nm) through the follicular pathway.

Contemporary studies advocate rapid and accurate treatment that hybrid the use of active compound with anti-inflammatory potential and efficient drug delivery modus. The application of nanocarriers assists in preferentially delivering the drug to the inflamed tissues in considerable concentrations due to the enhanced permeability and retention effect (EPR) and allow for controlled drug release within the targeted locations, which would significantly alleviate the need for frequent or continuous high doses of the drugs and eliminate off-sites side effects^10,11^.

Etodolac (ETD) is a preferential cyclooxygenase-2 (COX-2) inhibitor, non-steroidal anti-inflammatory drug (NSAID) that functions as a prostaglandin inhibitor at inflammation sites^12^. Interestingly, it exhibits a pH-dependent solubility, as its solubility dramatically increases at high pH and decreases as pH declines (pKa = 4.65). The clinical use of ETD as a conventional oral formulation fails to achieve the desired clinical effect because of its poor water solubility, limited bioavailability, in addition to its high protein binding and extensive metabolism (t_1/2_ = 6–8 h). As a result, frequent high doses of the ETD are required, increasing the risk of gastrointestinal, renal, and cardiac adverse effects and patient incompliance^13,14^.

The development of nanocarriers for the local release of high doses of the ETD at the inflammation site becomes of primary interest because it avoids hepatic first pass metabolism, improves stability and solubility of the ETD, and results in better patient compliance. So far, limited number of publications focus on the development of topical ETD formulations as an alternative route for oral administration^15,16^.

Natural lipids, such as phospholipids and triglycerides, can easily assemble stable dispersed nanovesicles with an oily outer phospholipid bilayer coat and inner aqueous core. These vesicles have the capacity of incorporating poorly soluble drugs in the oily layer and dispersing it in an aqueous medium by facing the outer surroundings with their hydrophilic heads, these improved drug permeation through the skin with considerable drug loading and stability. Liposomes, niosomes, emulsomes, ethosomes, and several other nanovesicles, such as glycerosomes and hyalurosomes comprising glycerol and hyaluronic acid, respectively, were designed and evaluated for skin delivery^17–19^.

Phosal® 53 MCT is a phosphatidylcholine (PC)-containing brand product. According to the manufacturing data sheet, Phosal® was found to contain a high PC concentration (approximately 59%), which is a natural lipid with good emulsification efficiency; thus, it was selected as the oily phase in addition to caprylic/capric triglyceride, oleic acid, ascorbyl palmitate, α-tocopherol, and glyceryl stearate. Caprylic/capric triglycerides are widely used in topical skin products, owing to its rapid penetration and softening ability. It builds up from medium chain triglycerides acting as dispersing agent^20^. Oleic acid is a mono-structured fatty acid and one of the powerful permeation enhancer. It acts by opening channels in the stratum corneum, that promotes the delivery of drugs to deeper epidermal and dermal skin layers where inflammation sites are present^21^. Ascorbyl palmitate, is an amphiphilic derivative of ascorbic acid, exhibits both anti-oxidant and self-assembling in aqueous medium properties. In addition to glyceryl stearate as an emulsifier^22,23^. α-Tocopherol (Vitamin E) is a non-enzymatic, widely used, topical antioxidant. It serves against lipid peroxidation of unsaturated fatty acids. At the same time, it is the most exogenous anti-oxidant present in our skin tissues, protecting against free-radical damage^24^. On reviewing former studies on Phosal®, it was found that Phosal® was applied limitedly in pharmaceutical development. Phosal® has been found to be very good solvent for lipophilic drugs, increasing their bioavailability. It is used in various topical and oral formulations, such as formulation of self-emulsifying dispersions of curcumin, lutein and genistein^25–27^. Utilization of multi-component product, such as Phosal® in building up nanovesicles for dermal applications have the superiority of gathering the benefits of all essential ingredients in one component; facilitating scaling up process.

As one of the most expressed adhesion receptors in inflammation, CD44, is expressed on leukocytes and parenchymal cells as trans-membrane, surface receptor. It plays a significant role in leukocyte recruitment, interactions between leukocytes and parenchymal cells, in addition to regulation of leukocyte and parenchymal cell function^28,29^; implying that CD44 is strongly recommended as a target for novel interventions in inflammatory disorders. Based on the fact that CD44 is a major receptor for hyaluronic acid (HA), our focus was to incorporate HA in the gelling matrix proposed in the current investigation was first due to the nontoxic and non-immunogenic characteristics of HA, in addition to its biocompatibility and biodegradability^30,31^. Secondly, to augment the anti-inflammatory effect of ETD via targeting the CD44 inflammatory receptors.

With due respect to ETD therapeutic potential, in the current research, we attempted to encapsulate ETD in lipid nanovesicles (Phosalosomes, PHs) with comprehensive process and formulation optimization procedures. Physicochemical properties of the optimized formulation were assessed in terms of particle dimension and drug loading efficiency, morphology, entrapment efficiency, FT-IR, and DSC. The rheological properties of PHs-HA gel, in vitro release, ex-vivo skin permeation, skin distribution test, skin tolerance test, and finally in vivo anti-inflammatory and analgesic studies on animal models were elaborated.

## Results

### Formulation and optimization of PHs

Since our work is focused on the development and characterization of PHs as a nano-carrier, the solubility of ETD in Phosal® as the main oily component and those of the three different aqueous rehydration media were checked to ensure the ETD solubility in the final dispersion. The ETD is a hydrophobic drug with poor water solubility that floats on the water surface. Its solubility in water is found to be as low as 0.113 ± 0.032 mg/mL; its solubility in the PBS (pH 4.7) is 2.66 ± 0.052 mg/mL, whereas its solubility in the PB (pH 5.5) is 0.674 ± 0.126 mg/mL. The solubility in Phosal® is found to be as high as 188.03 ± 8.84 mg/mL.

#### Preparation method

PHs prepared by EI were uniform in size (size of 184.7 ± 1.38 nm, PDI of 0.224 ± 0.013) and exhibited significantly (*P * < 0.05) high ζP (−44 mV). This is in accordance with a previous study, where liposomes prepared by EI method showed a small PS up to 184 ± 1.33 nm and a high negative ζP^32^. In contrast to the expected high stability of PHs produced by this method, PHs were irreproducible and coalesced together, thus showing large PS (858.8 ± 16.73 nm) with less uniform PS distribution after 24 h of preparation (0.522 ± 0.014). This was accompanied by a significant (*p* < 0.05) decrease in ζP (−0.941 ± 0.05). On the contrary, the PHs prepared by FH method showed highly reproducible, small-sized particles (160.9 ± 11.12 nm) and ζP of −27 ± 7.58 mV. These vesicles maintained their PS and distribution with no significant (*P* > 0.05) change with time (more than three months) compared to those prepared by EI. Based on this outcome, FH method was selected for the subsequent investigations. The level of significance among paired tested formulations was tested using Two-tailed t-test.

#### Size uniformity techniques

Firstly, application of homogenization technique for 10 min implied significant and considerably decrease (*P* < 0.05) by approximately 82.77% and 51.52% on PS and PDI, respectively, with no significant (*P* > 0.05) influence on ζP. Further increase in homogenization time to 15 min represented a significant (*P* < 0.05) influence on both PS and PDI; PS decreased to 160 ± 7.01 nm with PDI of 0.348 ± 2.45. Unfortunately, further increase in homogenization time to 30 min, revealed a dramatic decrease on ζP (−6.58 ± 1.01mV) was observed.

Similar trend was observed upon the increase in the homogenization speed, the PS was first decreased from 160.3 ± 7.01 nm (PHs 3) to 130.4 ± 1.92 nm (PHs 5), thereafter, the PS increased to 156.9 ± 2.06 nm (PHs 6), Table 1.

**Table 1:** Influence of formulation and process variables on the particle size, PDI, and zeta potential of various phosalosomal formulations.

Formula	Phosal Conc. (% v/v)	Homogeniz-ation time (min)	Homogenization speed (rpm)	Sonication time (min)	Surfactant type	Size (nm)	Zeta (mV)	PDI
PHs 1	3	**0**	4200	5		1260 ± 11.5	−35.5 ± 4.38	0.849 ± 0.133
PHs 2	3	**10**	4200	5		217.0 ± 4.67	−33.0 ± 5.67	0.421 ± 3.041
PHs 3	**3**	**15**	**4200**	**5**		160.3 ± 7.01	−31.3 ± 6.37	0.348 ± 2.450
PHs 4	3	**30**	4200	5		144.4 ± 0.46	−6.58 ± 1.01	0.205 ± 0.030
PHs 5	3	15	**6700**	5		130.4 ± 1.92	−9.76 ± 4.72	0.145 ± 0.011
PHs 6	3	15	**12,000**	5		156.9 ± 2.06	−0.44 ± 7.13	0.135 ± 0.016
PHs 7	3	15	4200	**15**		160.9 ± 11.12	−28.7 ± 07.58	0.317 ± 0.045
PHs 8	3	15	4200	**30**		169.1 ± 1.94	−29.2 ± 12.03	0.284 ± 0.020
PHs 9	**1.5**	15	4200	15		199.9 ± 9.95	−42.2 ± 4.03	0.507 ± 0.036
PHs 10	**5**	15	4200	15		135.0 ± 1.70	−22.0 ± 8.65	0.326 ± 0.047
PHs 12	3	15	4200	15	**Span**	185.0 ± 2.40	−49.1 ± 6.15	0.491 ± 0.001
PHs 13	3	15	4200	15	**Tween (Oil phase)**	133.3 ± 0.61	−16.5 ± 3.10	0.297 ± 0.007
PHs 14	3	15	4200	15	**Tween (Aqueous phase)**	269.0 ± 24.33	−22.1 ± 3.38	0.748 ± 0.065
PHs 15	3	15	4200	15	**Tween + poloxamer**	277.6 ± 12.15	−15.4 ± 4.04	0.368 ± 0.032
PHs 16	3	15	4200	15	**Tween + poloxamer + SDC**	148.8 ± 5.51	−19.2 ± 3.41	0.526 ± 0.082
PHs 17*^a^	3	15	4200	15	**Tween (Oil phase)**	264.5 ± 8.21	−32.6 ± 4.67	0.245 ± 4.86
PHs 18*^b^	3	15	4200	15	**Tween (Oil phase)**	304.3 ± 3.89	−35.1 ± 4.56	0.597 ± 1.06

* Contains 0.06%w/v ETD, ^a^Rehydration with PB 5.5, ^b^Rehydration with PBS 7.4.

The bold values reveal the changed variables.

*Alternatively*, although the increase in sonication time did not significantly affect the PS (*P* = 0.101), with a slight decrease in ζP from −31.3 to −29.2 mV, when the sonication time was increased to 30 min, a more homogenous dispersion was produced by decreasing the PDI to 0.284 ± 0.02 nm, Table 1. In respect of PDI, 15 min sonication was used in the subsequent formulations as the difference in the PDI of samples sonicated for 15 and 30 min-samples was not comparable to the time and energy consumed by the sonication step. The same finding was obtained with amphotericin B emulsomes, where 12 min was chosen as the optimum sonication time^33^.

To sum up, 15 min and 4200 rpm were picked up as the optimal homogenization time and speed. Sonication time was selected as 15 min.

#### Surfactant screening

The selection of proper surfactant or group of surfactants is made up on trial and error. A variety of surfactants with different HLB were tried and compared to plain PHs dispersion.

Depending on the PS, ζP and PDI, all formulations showed particles with small size, good ζP and PDI (Table 1). Smallest PS was produced when Tween 80 (HLB 15) was added to Phosal® in the first preparation step. Tween 80 has the privilege of being miscible with oil, addition of Tween 80 to PHs, showed a significant (*p* < 0.05) decrease in PS (133.3 ± 0.661 nm), ζP (−16.5 ± 3.10mV) and a uniform PDI (0.297 ± 0.007). Mixing Tween 80 with non-ionic surfactant poloxamer (HLB 22) showed a more reduction in ζP. Further Addition of SDC to the surfactant mixture, increased ζP (−19.2 ± 3.41mV) with a significant increase (*P* < 0.05) in PDI (0.526 ± 0.082).

Addition of Tween 80 to the outer aqueous phase (Tween out, PHs 14) significantly (*P* < 0.05) increased PS (269 ± 24.33 nm) and PDI (0.748 ± 0.065). Use of Span 20 (HLB 8.6) revealed a great and significant (*P <*0.05) influence on ζP, raising it to -49.1 ± 6.15 mV, Table 1.

However, upon storage either at 8 °C or at RT for two weeks, all formulations demonstrated either phase separation with noticeable oil droplets or aggregation and formation of a white layer except for the formulation prepared with tween 80 (Fig. S2, Supplementary Information).

Thus, Tween 80 was chosen in subsequent formulations and mixed with the oily component in the first step (Tween in). The level of significance among paired tested formulations was tested using Two-tailed t-test.

#### Rehydration media

The use of PB (pH5.5), PHs 17 produced vesicles with smaller PS (264.5 ± 8.21nm ), more uniform PDI = 0.245 ± 4.86, and with slight decrease in ζP (−32.6 ± 4.67 mV) than on using PBS (pH7.4), PHs 18 where PS was 304.3 ± 3.89nm, and PDI was 0.597 ± 1.06, Table 1.

#### Phosal® concentration

Formulation made up of 1.5% w/v Phosal® had PS of 199.9 ± 9.95 nm and PDI of 0.507 ± 0.036. However, PHs which contains 5% w/v Phosal® had a smaller PS of 135 ± 1.70 nm and PDI of 0.326.4 ± 0.047. The decrease in PDI was also observed in a previous study, when the amount of Phosal® was increased from 300 to 600 mg, PDI was decreased from 0.671 ± 0.027 to 0.502 ± 0.052, but with an observed increase in PS^34^.

On the PHs formulation, concentration of 3% v/v was the concentration of choice, to ensure homogenous dispersion (PDI = 0.317), and complete solubility of ETD in Phosal® based on its solubility (188.03 ± 8.84 mg/mL). The level of significance among paired tested formulations was tested using Two-tailed t-test.

#### ETD concentration

**Figure 1:**
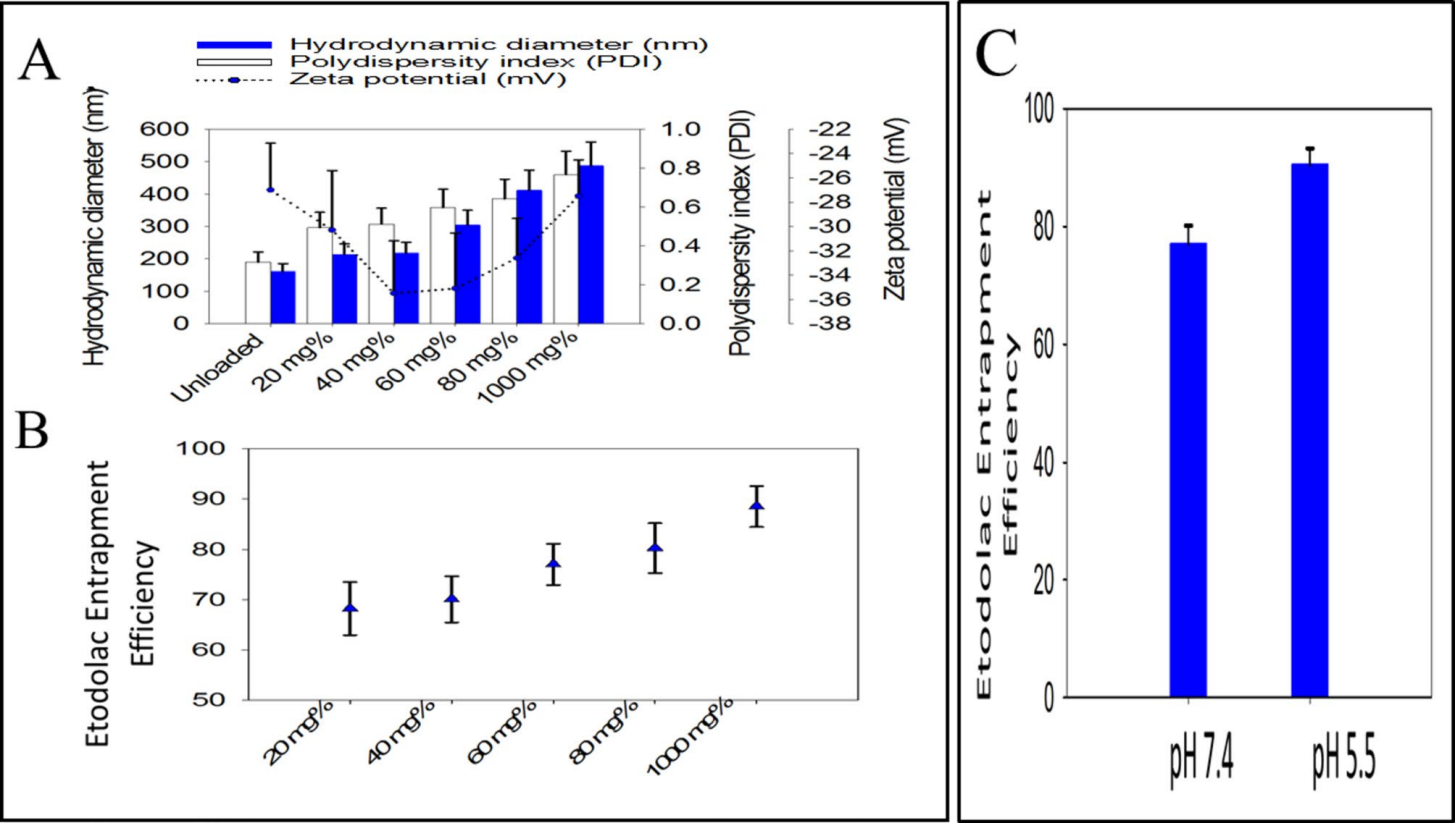
Effect of ETD concentration on (**A**) the particle size, zeta potential and polydispersity index, (**B**) % entrapment efficiency, (**C**) Effect of pH of rehydration media on the % entrapment efficiency, (GraphPad Prism 7.0, https://graphpad-prism.software.informer.com/7.0/#:~:text=GraphPad%20Prism%20combines%20scientific%20graphing,%2C%20statistics%2C%20and%20data%20organization.

Increasing the ETD loading showed a significant (*P* < 0.05) and noticeable influence on the all three parameters, Fig. 1A. PS (487.1 ± 1.41 nm) increased in case of 1% w/v ETD-PHs. The PDI were also significantly (*P* <0.05) increased as a function of the free unentrapped ETD.

Consequently, as a more uniform size was required, different size reduction methods were applied. Probe sonication showed the least and more uniform size (0.261 ± 0.036 nm) with unfortunately low zeta potential (−11.6 ± 3.03 mV). Since, the threshold of particles agglomeration ζP was from −11 to −20 mV. Increasing the sonication time to 30 min was used as a size reduction method to maintain a slightly higher zeta potential (−23.4 ± 1.31 mV) and to ensure a higher dispersion stability^35^. Increasing the sonication time to 30 min was chosen for its minimum effect on ζP. Sonication time of 30 min proved to have an influence on both PS and PDI. PS was decreased to 277.4 ± 6.98 nm and PDI was decreased to 0.416 ± 0.013.

### Physical and solid-state characterization of PHs

#### Drug content and encapsulation efficiency

The drug content in all formulations was in the range (74.15 ± 1.27%)–(84.93 ± 1.25%) of the theoretical initial loading. Regarding the EE%, all formulations demonstrated good EE% of ETD in the range 60–90%. It was observed that increasing the initial drug loading significantly increased the EE% (*P* < 0.05) (Fig. 1B), this finding matches that obtained by Alam et al.^36^. Figure 1C reveals that the use of PB (pH 5.5) in the rehydration, PHs 17, significantly (*P* < 0.05) increased the EE% compared to that prepared with the PBS (pH 7.4), PHs18, Table 1. The level of significance among paired tested formulations was tested using Two-tailed t-test.

#### Transmission and scanning electron microscopies (TEM and SEM)

**Figure 2:**
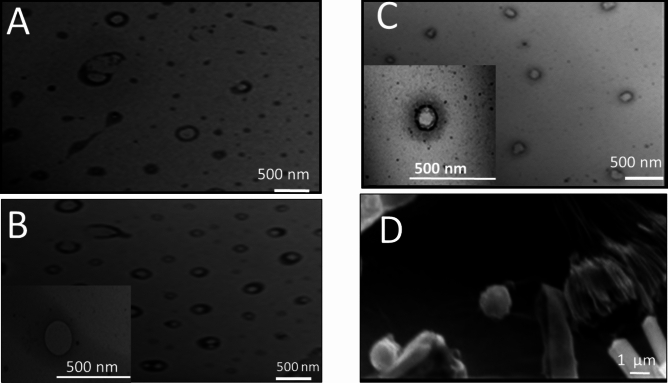
TEM image for (**A**) blank PHs prepared with PBS (pH7.4) by FH method (**B**) blank PHs prepared with PB (pH5.5) by FH method, (**C**) blank PHs prepared by EI method, and (**D**) SEM image of lyophilized PHs at magnification power of 7500. The scale bar in images A, B, and C is 500 nm and in image D is 1 µm.

All blank formulations photographed using TEM, showed spherically shaped vesicles, with an internal core and a lipid coat. Vesicles were in the nano-metric size and with no aggregation. PHs prepared by both methods (FH, EI) were of uniform small size, spherically shaped and of a core and shell structure. PHs prepared by FH method showed a perfect spherical shape with a uniform coat (Fig. 2A,B), while those prepared by EI method showed a core covered with channel-like coat that may be created as a consequence of ethanol evaporation (Fig. 2C).PHs prepared in PB (pH5.5) revealed a superiority in PS distribution, and a more uniform a shell-core structure rather than those prepared using PBS (pH7.4), Fig. 2B coherent with previous size measurements. SEM photograph of lyophilized optimized PHs formulation (Fig. 2D), reveals spherical particles that maintain its shape and consistency after lyophilization.

#### Fourier transform infrared (FT-IR)

**Figure 3:**
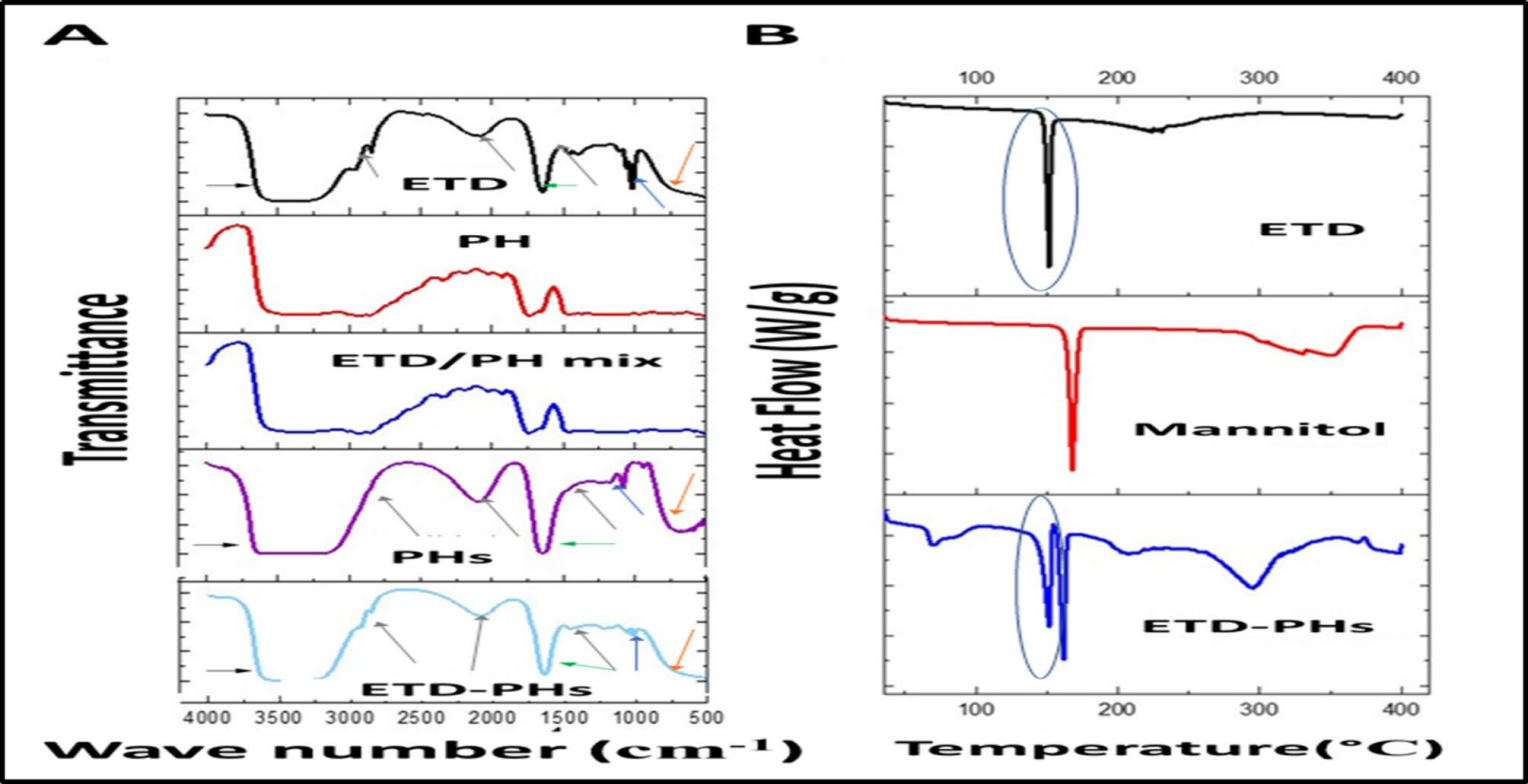
(**A**) FT-IR spectra of ETD, Phosal (PH), ETD/ phosal physical mixture (ETD/PH mix) and ETD loaded versus blank PHs formulations measured at 500–4000 cm^−1^. (**B**) DSC thermogram of Mannitol, ETD, and lyophilized ETD-loaded phosalosomes (ETD-PHs) measured at 35–200 °C.

The occurrence of any ETD interaction with other formulation ingredients was further predicted through the conduct of FT-IR studies. The spectra of the ETD, Phosal®, ETD/ Phosal® physical mixture, blank PHs, and ETD-PHs are shown in Fig. 3A. The different characteristic peaks of the ETD were specifically detected at 1032.2 cm^−1^ corresponding to –CO stretching vibration mode (blue arrows), 748 cm^−1^ corresponding to –NH wagging mode (orange arrows), a peak at 1744.2 cm^−1^ corresponding to the carbonyl group of the carboxylate stretching mode (green arrows), and 3530 cm^−1^ corresponding to the hydroxyl group of carboxylate mode (black arrows). Other peaks at 2600 cm^−1^, 2943 cm^−1^, and 1414 cm^−1^ corresponding to –CH3 asymmetric deformation mode (gray arrows) were also observed in the ETD/ Phosal® physical mixture and ETD-PHs spectra; these indicate no drug–PHs interaction^37^.

#### Differential scanning calorimetry (DSC)

The occurrence of any interaction between the ETD and other excipients as well as the state of drug within the formulation were investigated through the conduction of DSC studies. The thermograms of the pure drug, mannitol, and lyophilized PHs are shown in Fig. 3B. Melting endothermic peak of ETD with an onset temperature of 151.49 °C has been revealed by ETD powder thermogram; this indicates the crystalline nature of ETD. While, mannitol exhibited a peak at 164 °C, whereas the thermogram of lyophilized ETD-PHs have the same peaks at practically the same temperatures’ onset of 150.6 and 160.7 °C (Fig. 3B); the foregoing indicates the absence of ETD/formulation interactions and ETD was maintain its crystalline nature within PHs formulations^38^. Another endothermic peak was observed at 273.3 °C in the thermogram of lyophilized PHs that may be related to D-mannitol polymorphism, which may have two peaks on DSC analysis^39^.

### Gel formation and characterization

Evidently, the PHs dispersion has a low viscosity that hinders its dermal application. Therefore, engaging PHs in the gel formulation is recommended to increase skin contact and tp maximize their clinical effectiveness. Compared to liposomes, the phospholipid/HA combination (hyalurosome) has been previously observed as demonstrating high stability, better availability through the skin, and the capability to fast heal wound scratches^18^. Based on these interests, the ETD-PHs-HA gel was formulated and characterized in terms of its viscosity, spreadability, pH, and clarity. In comparing the plain HA gel, the HA gel containing free ETD (ETD-HA), and the ETD-PHs-HA, the results conferred that all formulations were clear, with an acidic pH values ranged 5.90–6.50. An acidic pH is highly suitable for skin application because of the acidic environment of the skin^40^.

**Figure 4:**
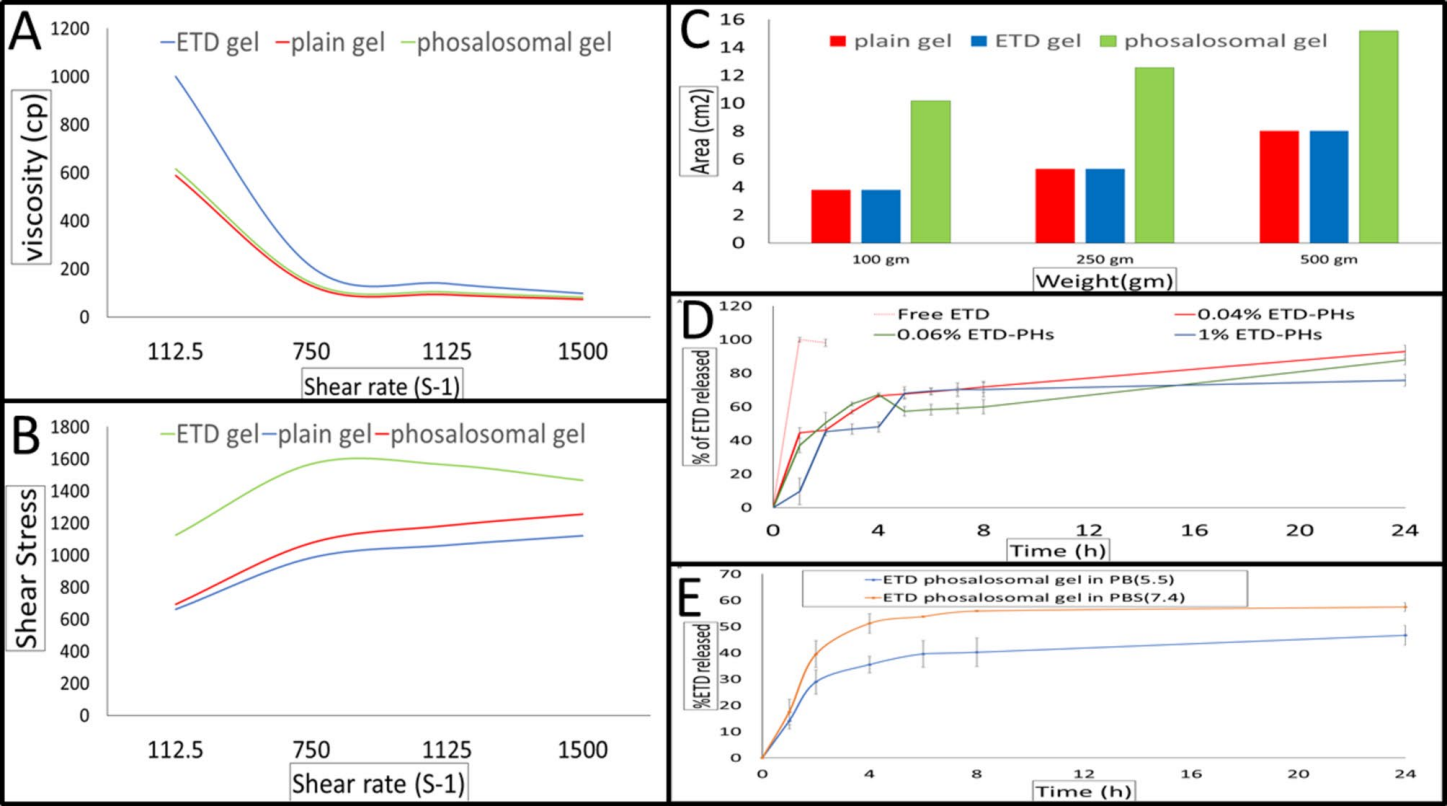
Characterization of ETD-PHs-HA gel, ETD loaded HA gel, and HA gel. (**A**) Shear stress vs. viscosity plot (**B**) Shear stress vs. shear rate plot; (**C**) Comparison of spreadability profiles at different weights (g), (**D**) In-vitro ETD release profiles from Phosalosomes formulations (ETD-PHs), with different loading concentrations compared to free drug in PB (pH 5.5), and (**E**) In-vitro ETD release profiles from ETD-loaded phosalosomes-HA (ETD-PHs-HA) gel in PBS (pH 7.4) and PB (pH5.5) at 32 ± 0.5 °C for 24 h using dialysis-bag method.

The viscosity of the three gel formulations exhibited a shear-thinning behavior; as viscosity decreased with increasing shear rate (Fig. 4A). In addition, non-Newtonian behavior was observed because there was no linear correlation between shear stress (Pa) and shear rate (s^−1^) (Fig. 4B); this indicates the presence of pseudo-plastic flow. Such a flow is common among topically applied formulations, where the resistance of the gel to flow decreased with the application of shear stress^41^. The spreadability of ETD-PHs-HA gel was higher than those exhibited by both plain HA gel, HA gel containing free ETD (Fig. 4C).

The spreadability of ETD-PHs-HA gel was the highest and may be related to the presence of PHs within the gel system that reduced the gel flow resistance^42^.

### In vitro release assessment

From the presented plots (Fig. 4D), it was found that all formulations have controlled ETD release over 24 h compared to the ETD solution. The PHs formulations exhibited a burst initial drug release, and this may be attributed to the unentrapped drug that partitioned immediately and diffused to the release medium. As the initial drug loading increased to 1%w/v, T_50_ was significantly increased (approximately 4.5 h).

Furthermore, the release of ETD from PHs-HA gel was tested in both PBS (pH7.4) and PB (pH5.5) to mimic the skin acidic conditions and represented in (Fig. 4E). ETD release pattern from PHs-HA gel in PBS (pH7.4) exhibited a T_50_ that is practically the same as in case of PHs dispersion. After 4 h, ETD-PHs-HA gel exhibited a slower and more controlled release compared to PHs dispersion with only 57.49 ± 1.6% ETD released after 24 h. The release pattern in PB (pH 5.5) was slower and more controlled than PBS (pH 4.7). T_50_ was increased from ~ 4 h in PBS (pH 7.4) to more than 8 h and only 46.66 ± 3.80% ETD released after 24h.

### Ex-vivo permeation and retention study

**Figure 5:**
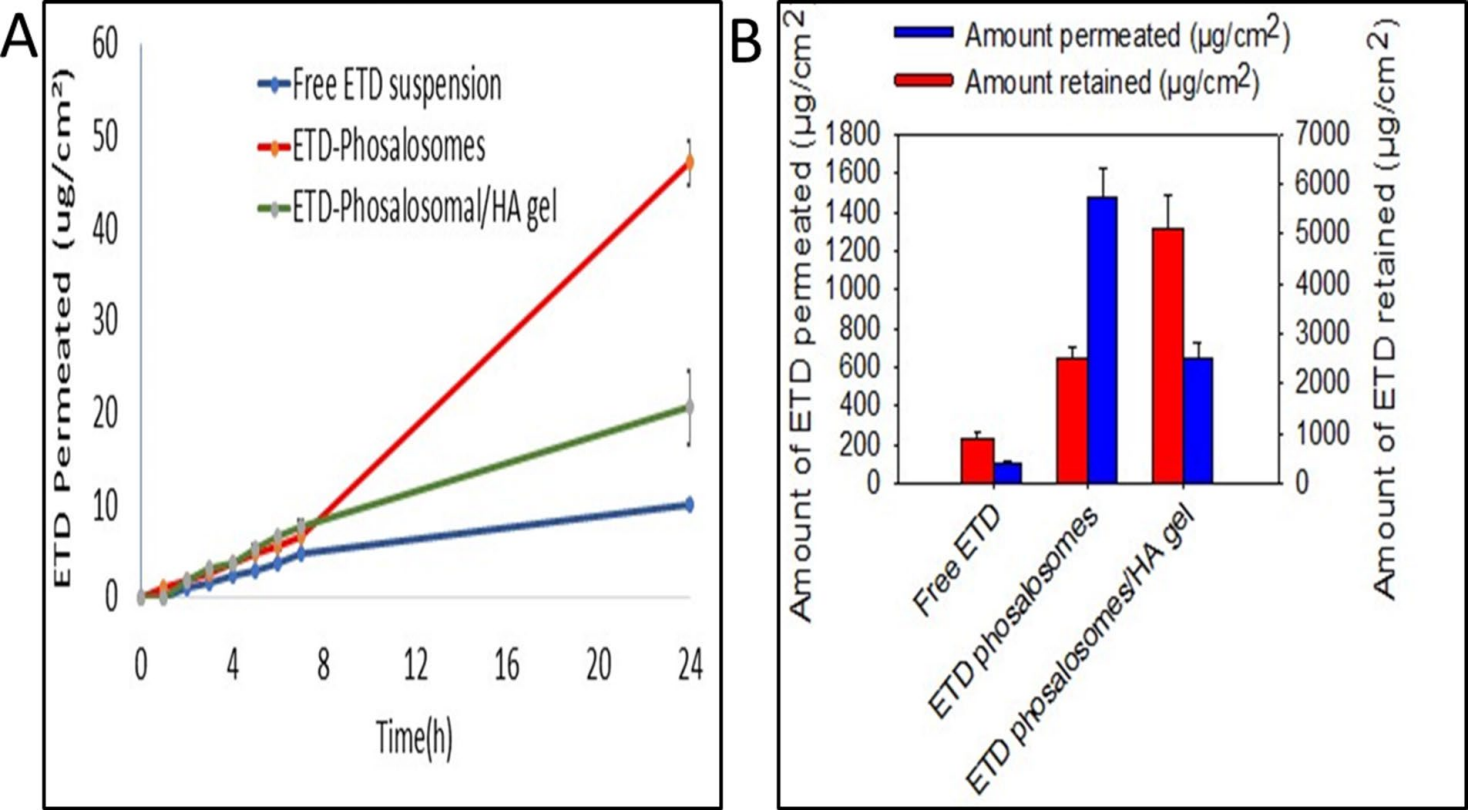
(**A**) Cumulative amount (µg/cm2)-time profiles of ETD permeated across rat skin from Free ETD suspension, ETD-PHs, and ETD-PHs-HA gel, using Franz diffusion cell. (**B**) Cumulative amount of ETD (µg/cm2) permeated and retained in rat skin following application of the three different formulations after 24 h. Each value stands for the mean ± SD (n = 3).

All formulations showed significant differences in the amount of ETD permeated along the experiment time (Fig. 5A), with significant (*P* < 0.05) differences in their lag times (Table 2). With the use of ETD-PHs dispersion, the lag time was minimum (0.33 h), while the highest (1.014 h) was attained when ETD-suspension was used. This observation was augmented by the significant (*P* < 0.05) increase in Jss (5.53 µg/cm^2^ h) upon the incorporation of ETD into PHs compared that in the ETD suspension (0.095 µg/cm^2^ h). As recorded in Table 2, the highest diffusion coefficient returns to ETD-PHs (6.389*10^–11^ cm^2^/s) with lowest surface concentration compared to ETD suspension (4.564*10^–11^ cm^2^/s). Upon incorporation into HA, ETD-PHs-HA lag time increased to 0.63 h. Moreover, the percentage of ETD permeated from PHs dispersion after 24 h was about 2.5-folds higher than that from ETD-PHs-HA gel formulation and only 5.35% of ETD permeated after 24 h from ETD suspension (Fig. 5B). This could be attributed to the lipid-facilitating permeation ability in addition to the enhanced ETD solubility after incorporation into PHs. However, longer lag time in case of ETD-PHs-HA was expected because of the time required for the drug to diffuse through the polymer matrix before its permeation to the skin.

**Table 2:** Calculated permeation parameters of ETD suspension, ETD Phosalosomes (ETD-PHs) and ETD phosalosomes- HA (ETD-PHs-HA) gel.

Sample	Flux (µg/cm^2^.h)	Lag time (h)	Skin surface concentration (C_0_, µg/cm^3^)	Diffusion coefficient (D, cm^2^/s)
ETD suspension	0.868	1.014	5.284*10^3^	4.564*10^–11^
ETD-PHs	1.111	0.333	5.221*10^3^	6.389*10^–11^
ETD-PHs-HA gel	1.374	0.639	5.274*10^3^	7.237*10^–11^

The amount of drug localized in the skin significantly increased with the incorporation of ETD into PHs and further in HA gel. Incorporation of loaded PHs into HA gel increased the amount retained to 511.89 ± 62.65 µg/cm^2^ compared to 249.47 ± 93.97 µg/cm^2^ and 91.01 ± 48.32 µg/cm^2^ for ETD-PHs dispersion and ETD suspension, respectively. The best results were achieved by the HA gel with a moderately small amount permeated (10.95%) and the most proportion localized in the skin (60.31%), which is our target in this research, Fig. 5B. The minimization of transdermal permeation and systemic absorption are of the fundamental for dermal therapy. In general, the permeated amounts of ETD are considered small, so that there is no concern for severe systemic side effects. The level of significance among paired tested formulations was tested using Two-tailed t-test.

### Visualization of dermal distribution

**Figure 6:**
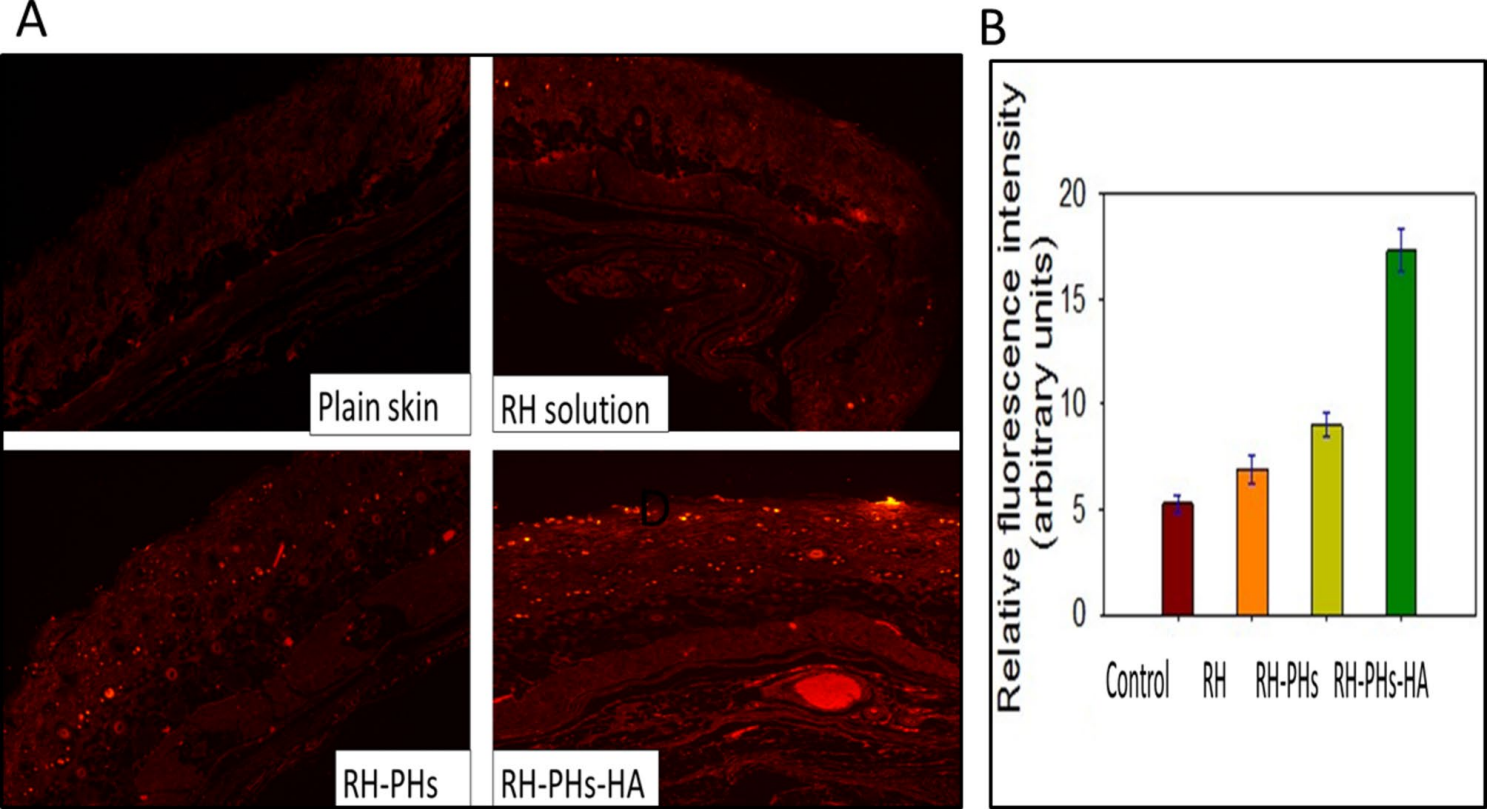
(**A**) Fluorescence microscopic photographs of dermal distribution of fluorescent dye (Rhodamine, RH) in plain skin with no RH, skin treated with free RH solution, skin treated with RH-PHs, and skin treated with RH-PHs-HA gel. (**B**) The calculated relative fluorescence intensity of different RH formulations in comparison to the normal skin image (control).

Fluorescence microscopy was applied to analyze the skin localization of formulation and ETD penetration pathway. A fluorescent dye (RH) dermal distribution experiment was performed on rat dorsal skin to determine the skin penetration potential of RH-PHs, RH-PHs-HA gel, and RH solution (Fig. 6A). Accordingly, RH was chosen as a dye because its Log P value and molecular weight are 3.1 and 479.02 g (defined by distributer) and those of ETD are of 4.65 and 287.35 g, respectively. ETD is more hydrophobic and has a smaller molecular weight and a larger partition coefficient; these ensure better skin behavior^13^. Hence, RH was selected for investigating the distribution across all skin layers. Compared with the RH solution, the incorporation of RH in PHs dispersion and furtherly into PHs-HA gel resulted in an improved penetration and more homogenous distribution through different skin layers with greater localization, compared to normal skin as the negative control (Fig. 6A).

The calculated relative fluorescence intensity of RH permeated from PHs-HA was threefold and 2- fold higher than RH solution and to PHs, respectively, Fig. 6B. Most of the permeated dye accumulated in the ducts of hair follicles and sweat ducts as revealed in the fluorescent images.

### Quantitative bio-distribution studies

**Figure 7:**
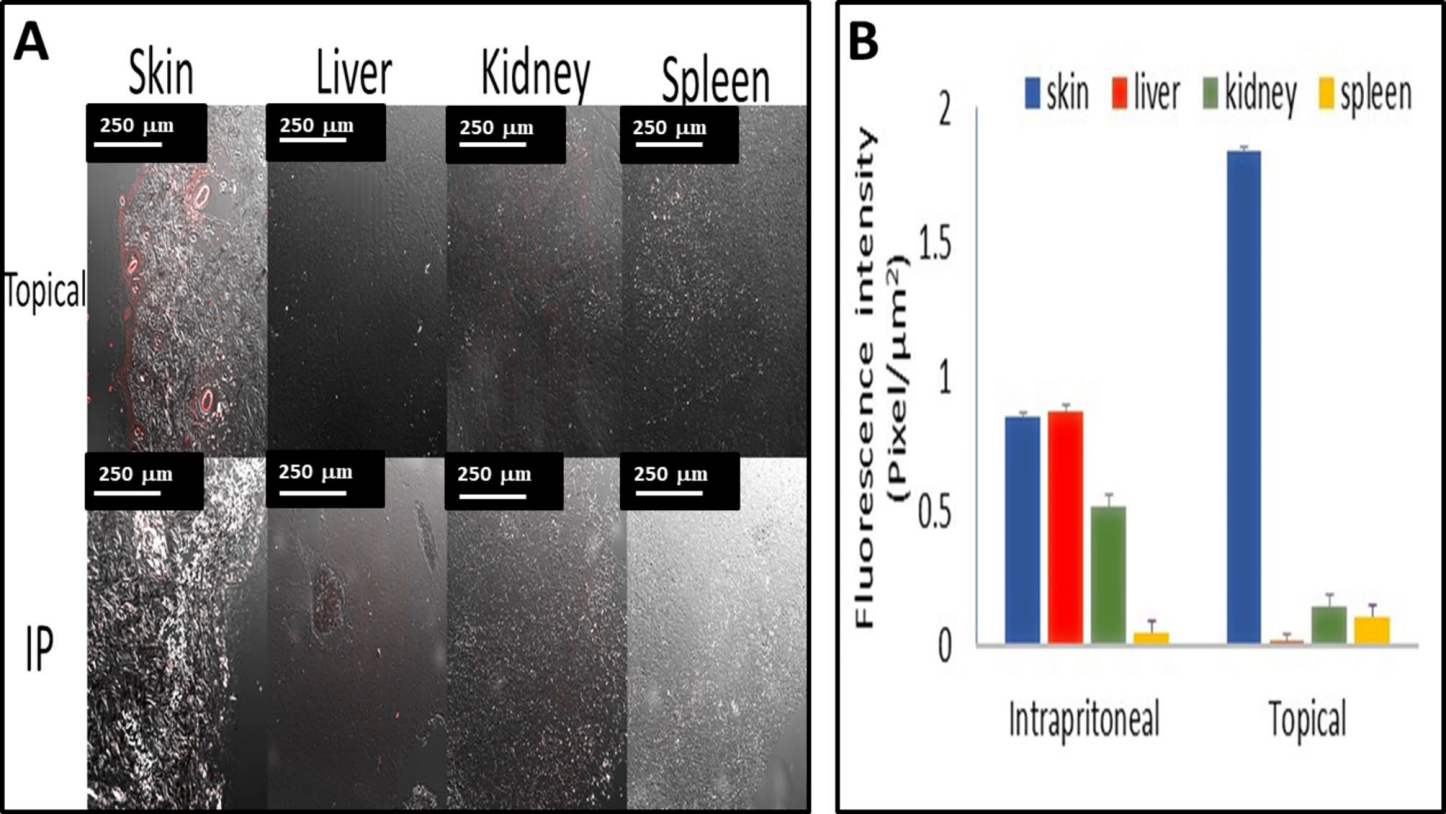
(**A**) Confocal Laser Scanning Microscopy (CLSM) cross-sectional images of organs; skin, liver, kidney, and spleen with fluorescence probe after topical and intraperitoneal applications of RH dye (**B**) Quantification of fluorescence intensity (Pixel/µm^2^) for CLSM images of organs; skin, liver, kidney after intraperitoneal and topical doses.

By the aid of the confocal laser microscopic photographs, it is clear that topical dose was significantly localized in the skin stratum corneum, epidermis; thereafter they diffused through the dermis and around the ducts with 2.5 folds higher than that following the IP dose. The deposition of RH dye after topical application, was seen to concentrate in the hair ducts, thus emphasizes the trans-appendageal route as the main route for PHs permeation, Fig. 7A.

On the contrary, the RH quantified in the liver and kidney following topical administration; 29 and 5.7 folds lower than IP dose, Fig. 7B. These observations verify our main goal, which is the localization of ETD at the site of action in addition to decreasing its toxicity by minimizing its accumulation in other vital organs.

### In vivo skin irritation test

**Figure 8:**
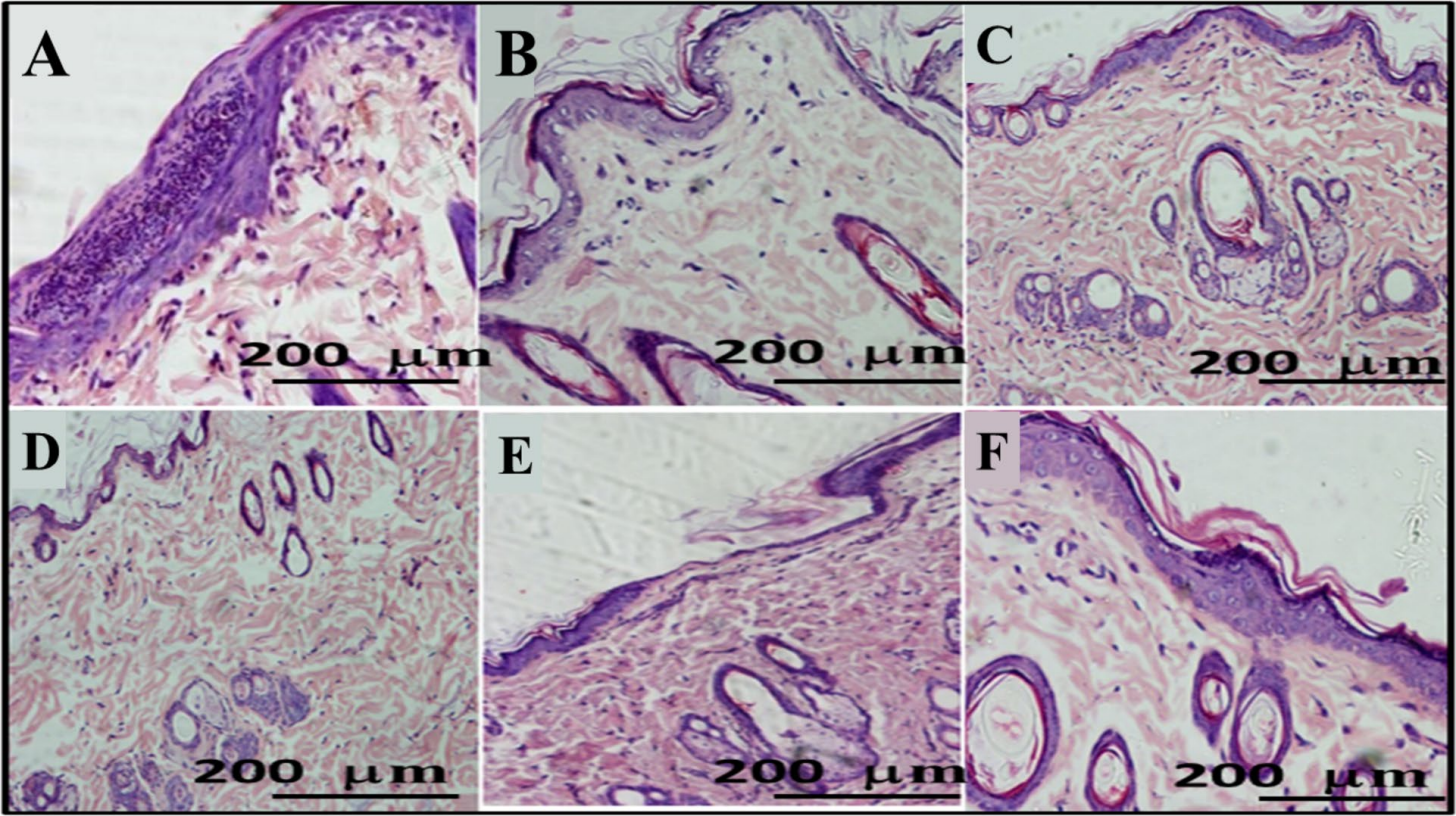
H and E staining of skin sections for skin compliance test: (**A**) inflamed skin (+ ve control), (**B**) normal skin (−ve control), (**C**) ETD solution (one dose), (**D**) ETD PHs-HA gel (one dose), (**E**) ETD solution (successive doses) and (**F**) ETD-PHs-HA gel (successive doses). Each image represents respective treatment group (n = 3).

The skin compliance is a cornerstone in preparation of topical drug delivery systems. It was performed to determine the compatibility of free ETD solution and ETD-PHs-HA gel on rat skin after application of one and successive doses. The data obtained demonstrated no marked visual dermal changes such as erythema during the test period. Histological examination of rat skin treated with phenol as a positive control showed hyperkeratosis with a complete loss of filaments (Fig. 8A). Rat skin treated with a single dose of ETD solution (Fig. 8C) and ETD-PHs-HA gel (Fig. 8D) showed an intact stratum corneum and the subsequent epidermal layers, with no evident change in histopathological features and comparable to normal skin (Fig. 8B).

Furthermore, after successive doses, ETD solution showed thinning in the epidermis with the infiltration of stroma (Fig. 8E). On the other hand, a homogenous epidermis with no inflammatory symptoms (thinning or thickening) and with normal stratum corneum and filaments were observed in skin exposed to ETD-PHs-HA gel (Fig. 8F) compared to normal skin (Fig. 8B); these indicate that the designed gel formulation is safe for topical application.

### In vivo anti-inflammatory and antinociception potential

It is noteworthy that the ETD is indicated for the management of rheumatoid arthritis, osteoarthritis and juvenile rheumatoid arthritis as an anti-inflammatory drug and as a potent analgesic either for acute or chronic pain; it is well tolerated compared with other NSAIDs^43^.

The efficacy of ETD- PHs-HA gel formulation as an anti-inflammatory and analgesic drug have to be proven by the decrease in inflammation in rat ears as well as the delay and decrease in the licking time of rat paw.

#### Acute anti-inflammatory test (Anti-edema)

**Figure 9:**
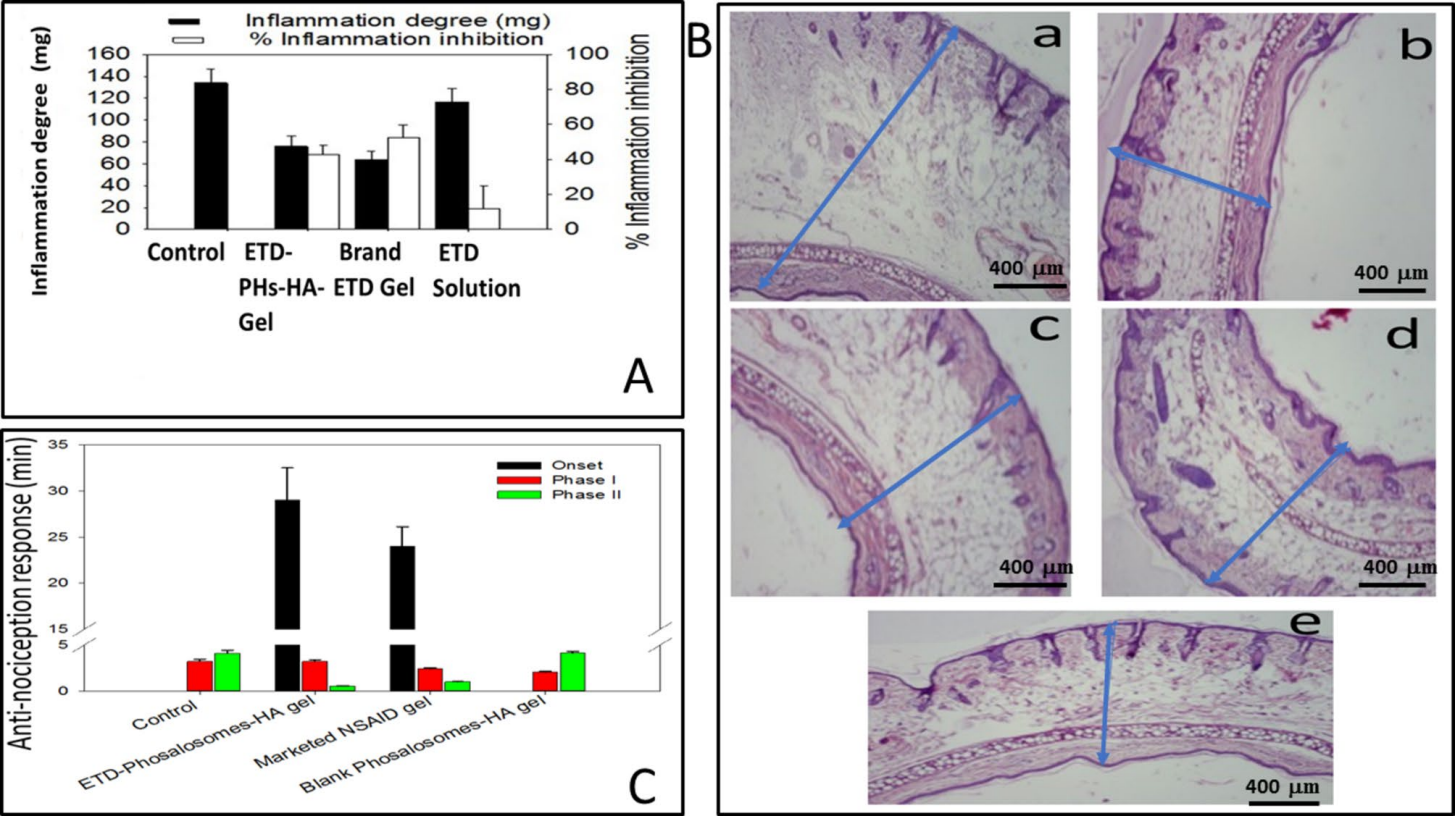
(**A**) In-vivo anti-inflammatory potential in rats treated with 5% w/v phenol. Control untreated rats were compared with those treated with ETD-Phosalosomes-HA (ETD-PHs-HA) gel, marketed NSAID gel and ETD-solution, (**B**) Histopathological skin examination of anti-edema test: (**a**) inflamed skin, (**b**) normal skin, (**c**) skin treated with ETD solution, (**d**) skin treated with ETD-PHs -HA gel and (**e**) skin treated with marketed NSAID gel, and (**C**) In-vivo anti-nociception response in rats treated with 5% v/v formalin. Control untreated rats were compared with those treated with blank and ETD loaded Phosalosomes-HA (ETD-PHs-HA) gel as well as marketed NSAID gel.

To study the anti-inflammatory effect of the prepared ETD formulations, phenol was applied to the rat ears; this resulted in their spontaneous inflammation and size enlargement. In vivo results were expressed in terms of the average increase in ear thickness. The percentage of inhibition in ear edema and inflammatory degree as a measure of average increase in ear weight are presented in Fig. 9A. Subcutaneous injection of phenol in the dorsal surface of the right ear (20 µL per ear) resulted in a 61% increase in the ear weight, compared to the normal ear. Ear treated with ETD-PHs-HA exhibited an inflammatory degree of 76 ± 0.004 mg compared to 116.5 ± 0.009 mg for ears treated with ETD solution, and 134 ± 0.011 mg for untreated group (Fig. 9A).

ETD solution accounted for only 11.83 ± 0.29% inhibition of edema. The addition of ETD to PHs-HA significantly increased the anti-edema activity in the animal model; this resulted in an inhibition percentage of 42.92 ± 0.07% and the implementation of the anti-inflammatory effect of ETD, Fig. 9A.

As presented in Fig. 9B (The optical microscopic photographs), no remarkable reduction in thickness upon using ETD solution, Fig. 9B-c (*P* > 0.05) compared to positive control group, Fig. 9B-a (456.16 ± 25.48 and 516.06 ± 20.58 µm, respectively). Controversy, there was a significant(*P* < 0.05) and dominant reduction upon using ETD-PHs-HA gel to be 404.53 ± 11.54 µm, (Fig. 9B-d) and marketed product (394.83 ± 18.05 µm, Fig. 9B-e), when compared with positive control group (Fig. 9B-a) and plain skin as a negative control (319.83 ± 13.18 µm, Fig. 9B-b). Results were highly comparable to that yielded by the marketed product and confirms the anti-inflammatory potential of the formulations.

A previous comparative study on diclofenac as non-selective COX inhibitor was elaborated, the diclofenac nanosuspension demonstrated a higher accumulation of diclofenac in the skin with a 50% inhibition of in vivo edema compared to both conventional suspensions and the commercial formulations^44^. The level of significance among paired tested formulations was tested using Two-tailed t-test.

#### Anti-Nociception test

The formalin injection test is preferably used as an injury-produced pain model. Upon the injection of formalin into the rats, a biphasic nociception response is observed. It was characterized by an early phase, which started directly after administration (0–10 min) and as a response to the stimulated peripheral nociceptors; it lasted for few min followed by a quiescent interval.

Thereafter, a second more characteristic phase (20–40 min later) followed; it lasted for a few minutes as a response to the inflammatory pain^45^. Figure 9C summarizes the results and reveals that ETD-PHs- HA gel and the marketed product exhibited a noticeable delay in the onset of the first phase for 30 min with no response.

It was followed by the first licking phase, which lasted for 3:20 ± 0.039 and 2:40 ± 0.013 min for ETD-PHs-HA and brand product, respectively.

Thereafter, a second phase lasted for significantly short period (less than 1 min) for both groups. The results were compared with the untreated group and group treated with blank PHs-HA gel (4 min). In both groups; untreated and treated with blank PHs-HA gel, rats started licking immediately after formalin injection. Untreated group showed two phases of licking lasted for 3:19 ± 0.018 and 4:07 ± 0.003 min, while group treated with ETD solution showed 2:03 ± 0.048 and 4:10 ± 0.10 min phases.

Since, ETD and the marketed product were COX-2 inhibitors, which inhibited PGs production. There is no difference between ETD-PHs-HA and the marketed product with the untreated group in the first phase (histamine and serotonin mediated). In PG-mediated second phase, a significant reduction in the licking time was observed.

### Stability study

Stability of ETD PHs dispersion and gel was studied over a three-month period. The physio-chemical stability of the dispersion was assessed by determining any change in PS, PDI, and ζP as well as ETD release profile from ETD-PHs dispersion compared to the freshly prepared dispersion. Formulations were stored at 2–8 °C and protected from light. After the storage period of three months, no particle aggregation was observed through visual inspection.

In measuring other parameters, no statistical significant differences (*P* > 0.05) were observed in the particle size, ζP and PDI (Table S1, Supplementary Information); this indicates the stability of PHs particles in dispersion with no aggregation over the test period. Figure S3-A, (Supplementary Information) represents the release profile of ETD from freshly prepared PHs and three- month stored PHs. The stored PHs showed more slow and controlled release profile than fresh PHs.

Regarding the stability of ETD-PHs-HA gel, it maintained its uniformity, homogenous distribution, and consistency till more than three months (Fig. S3-B-c, Supplementary Information). On the other hand, HA gel loaded with free-ETD (Fig. S3-B-b, Supplementary Information), showed drug crystals precipitation after three months. This finding emphasizes the real impact of PHs in enhancing drug dispersibility and finally drug stability within the HA gel matrix.

## Discussion

The design of the PHs structure enabled the lipophilic ETD to be localized in the phospholipid bilayer and stably dispersed in the aqueous medium. The same results were obtained when the solubility of poorly soluble curcumin was considerably increased by 10,000 folds when incorporated in curcumin emulsomes; this formed a homogenously dispersed colloidal solution in water^46^. Several preliminary parameters were assessed for optimization.

To prepare versatile lipid bilayer-coated vesicles, different methods (such as thin-film hydration, ethanol injection, reverse-phase evaporation, and double emulsion methods) have been utilized. The film hydration (FH) method is one of the most widely used and simple techniques to prepare a diversity of nanocarriers, such as liposomes, emulsomes, and niosomes^47^. In the FH method, after the complete evaporation of organic solvent under reduced pressure, the lipid was casted as a thin film. Upon rehydration and manual shaking, the lipid swelled and formed a dispersion of large unilamellar vesicles. Upon homogenization and further sonication, large unilamellar vesicles were transformed into small multilamellar vesicles. In the ethanol injection (EI) method, the ethanolic lipid solution was injected into the aqueous medium through a needle with subsequent ethanol evaporation. This immediate dilution with aqueous phase resulted in the precipitation of the lipid molecules forming bilayer fragments, which further lead to vesicles formation^48^. Cholesterol (CH) was used as a stabilizer in a concentration equivalent to 4% PC as Pal et al.^49^ recommended to be the optimum concentration. Pal et al.^49^ and Alam et al.^36^ proved that increasing the CH concentration can disturb the bilayer membrane structure; thus decreasing the drug EE%. Therefore, at this stage of investigation it can be stated that FH method would be utilized using CH as stabilizer. As stated in literature, when PS is in the range of 1nm-1µm, the preparation named colloidal dispersion. PHs were prepared as a white colloidal dispersion with PS less than 300 nm. Small PS is considered as a vital requirement for the development of topical formulations. The decrease in PS is accompanied by the increase in the surface area. Consequently, this increases the partitioning and further drug permeation through different skin layers. Verma et al.^50^ reported the formulation of liposomal vesicles ≤ 300 nm in size and demonstrated the ability of these vesicles to deliver the incorporated drug mostly into the deeper skin layers. Noteworthy, the obtainment of considerably small PS and high ζP, was an integral scope of the presented research. Negatively charged nanocarriers were found to permeate easily through skin layers. The repulsive forces that created between the nanocarriers and skin lipids with similar charges resulted in the production of channels through which drug can permeate^51^. To achieve such goals, different factors were investigated. Firstly, application of homogenization technique, this could be explained as M. Nakach et al.^52^ reported. They claimed that on high-energy milling, such as milling for long time or high speed, the PS started to decrease to some critical values. Any further energy supply to these particles lead to particles deformation as a result of energy accumulation on the surface of particles, and subsequently particles aggregation; known as negative milling phenomenon. The obtained data proved that homogenization was highly efficient in forming small emulsion droplets with homogenous distribution. Our results were in accordance with previously reported data, where the size of doxorubicin liposomes was reduced as the homogenization speed increased from 4000 to 10,000 rpm^53^.

When Phosal® was used solely in the preparation depending on the endogenous emulsification power of lecithin and glyceryl stearate, phase separation was observed with white supernatant layer, when the dispersion kept in both RT and 4 ºC that necessitate the use of external surfactant. As it was known by DLVO theory to ensure emulsion stability, all particles should be covered by the emulsifier. The increase in the repulsion force between the particles, increases the emulsion stability. Based on that, it can be concluded that lecithin and glyceryl stearate did not perfectly cover the particles surface, hence they gave the chance for particle aggregation and phase separation. The selection of proper surfactant or group of surfactants is made up on trial and error. A variety of surfactants with different HLB were tried and compared to plain PHs dispersion.

Smallest PS was produced when Tween 80 was added to Phosal® in the first preparation step. Tween 80 has the privilege of being miscible with oil, addition of Tween 80 to This could be explained on the basis of using lecithin and non-ionic surfactants in formulation, non-ionic surfactant doesn’t ionized decreasing ζP and intercalate itself between phospholipids, producing well packed mixed bilayer film with small and uniform PS^54^.

Addition of Tween 80 to the outer aqueous phase (Tween out, PHs 14) significantly (*P* < 0.05) increased PS and PDI. This may be explained by the assumption that one portion of Tween 80 was located between the oily and the aqueous components, allowing emulsification. The other portion was adsorbed on the surface; this increased the PS and ζP. However, upon storage either at 8 °C or at RT for two weeks, Tween 80 was tried as a single external surfactant, prolonged stability under both temperatures was observed for several months. Tween 80 with its ethylene oxides and hydrocarbon chains can aligned itself between hydrophilic and lipophilic domains. Tween 80 was reported to be safe and recommended for topical application, because it enhanced drug permeation through the skin^42^. Goindi et al.^16^ reported the highest solubility of ETD (162.72 ± 0.005 mg/mL) in Tween 80, compared to other surfactants.

Upon the use of PBS (pH7.4) for rehydration of 0.60 mg % loaded PHs, the dry lipid film was rapidly rehydrated and completely re-dispersed within an hour, the redispersion process was complete and efficient. Alternatively, upon the use of distilled water, the rehydration process was considerably difficult even after 24 h of soaking. In the case of PB (pH 5.5), the rehydration was efficient and complete after six h of soaking. This may be attributed to the higher solubility of ETD in PBS (pH7.4) than in the two other media. The higher solubility of ETD in PBS (pH7.4) promote the incorporation of ETD in the core, this increased the nanovesicle PS and changed the PDI. On the hand, lower solubility of ETD in PB (pH 5.5) allowed the presence of ETD in its unionized state, so more ETD was solubilized in lipid bilayer and minimum amount in aqueous core, giving rise to smaller PS and more uniform distribution. The increase of Phosal® concentrations in the aqueous phase, increased the proportion of PC. Increased PC constituted an enhancement in the viscosity of the dispersion and accompanied with a decrease in PDI^34^.

Increasing the ETD loading showed a significant and noticeable influence on the all three parameters and could be attributed to the increased incorporation of ETD and its solubilization in the lipid coat and aqueous core increasing PS, increased PDI as a function of the free unentrapped ETD. The increase in ζP was also confirmed by Lúcio et al.^55^ they suggested the influence of concentration-dependency of ETD on bilayer potential. Influence of pH-dependency of the ETD solubility on its EE% was observed. Since pKa is a measure of the extent of drug ionization, according to the pKa value of ETD (4.76), the drug was expected to be partially ionized when PBS (pH 7.4) is used. In the PB (pH 5.5), the drug is expected to be mostly in its unionized, lipid soluble form and suspected to be preferentially present in the oily phospholipid bilayer, thereby increasing its entrapment.

Formulation of HA gel has many privileges over other polymers used. As formerly described, HA is considered as a target ligand to CD44 which is one of the main inflammation cell adhesion receptors^28^. Moreover, HA with its high molecular weight and its high hydrophilicity, demonstrated the ability to surround its random-coil confirmation with water molecules (hydrogen bonds), resulting in a homogenous, viscous, and elastic gel^31^.

To evaluate the release pattern of ETD in the biological fluid at the site of action, the in vitro release test must be elucidated before drug-skin permeation, in order to feature the influence of the formulation composition on the diffusion pattern. Moreover, the impact of increasing ETD concentration in PHs, gel incorporation and the pH of release media were assessed. The PHs formulations exhibited a burst initial drug release, and this may be attributed to the unentrapped drug that partitioned immediately and diffused to the release medium. ETD release pattern from PHs-HA gel in PBS (pH7.4) exhibited a T_50_ that is practically the same as in case of PHs dispersion. Because in the preparation of HA gel, PHs dispersion was used without the separation of unentrapped drug. This free drug was dispersed within the polymer matrix and accounts for fast release. Moreover, stirring during gel formation and leaving for 24h may result in more ETD to be released in the external polymer matrix that can easily be diffused and can account for high T_50_. Thereafter 4 h, ETD-PHs-HA gel exhibited a slower and more controlled release compared to PHs dispersion. This may be attributed to the diffusivity of ETD through the polymer matrix, which decreased the rate and the cumulative amount of ETD released. The release pattern in PB (pH 5.5) was slower and more controlled than PBS (pH 4.7). This may be attributed to the lower solubility of ETD in PB (pH 5.5). Several studies confirmed that nanovesicles loaded gel has the potential to sustain drug release over prolonged time^40,42^.

The Higher percentage of ETD permeated from PHs dispersion after 24 h compared to ETD-PHs-HA gel formulation and ETD suspension could be accredited to the lipid-facilitating permeation ability in addition to the enhanced ETD solubility after incorporation into PHs. However, longer lag time in case of ETD-PHS-HA was expected because of the time required for the drug to diffuse through the polymer matrix before its permeation to the skin. Various factors, such as the hydrophobic nature, partition coefficient and molecular weight have exhibited an influence on the dermal penetration of drugs^13^. Accordingly, RH was chosen as a dye because its Log P value and molecular weight are 3.1 and 479.02 g (defined by distributer) and those of ETD are of 4.65 and 287.35 g, respectively. ETD is more hydrophobic and has a smaller molecular weight and a larger partition coefficient; these ensure better skin behavior^13^. The improved gel penetration and more homogenous distribution through different skin layers with greater localization may be related to the penetration enhancement effect of lipids used in the PHs formulation and their small size. Because the composition of any formulation affects its skin penetration by altering skin rigidity and hence determines its trans-epidermal or trans-follicular penetration capability. Moreover, the potential of HA to maintain the skin in a hydrated state, which affects both drug diffusion and partitioning into skin layers. This finding supports the retention results obtained formerly by Jain et al.^42^, who found that the incorporation of loaded Fluorescein isothiocyanate dye into lipospheres and subsequently into gel formulation promoted its penetration and distribution through different skin layers. Most of the permeated dye accumulated in the ducts of hair follicles and sweat ducts as revealed in the fluorescent images; this emphasizes the trans-follicular penetration as the dominant route. Many previous studies claimed the potential of liposome to target the skin appendages, as hair follicles and sebaceous glands. Plessis et al.^56^ observed the highest accumulation of liposomes loaded with γ-interferon in hamster skin with high follicular density compared to hairless skin, suggesting that the trans-follicular pathway could be the main route for drug deposition from liposome. Li et al.^57^ developed two types of flexible liposomes for antigen delivery, composed of phospholipids S 100 and CH. Thirty min after the topical application, particles were found to accumulate in the hair follicles and sweat ducts.

There was a considerable disagreement regarding the role of COX-2 expression in nociception. Certain studies suggested that COX-2 up-regulation requires hours after the injury^45^. Other investigations proved that COX-2 expression process is constitutive and imparts to rat skin normal physiology, thus selective COX-2 inhibitors could produce a significant anti-nociception in the formalin test. Diclofenac was found to produce a dose-dependent antinociceptive effect during phase two, but not during phase one^58^. Numerous clinical studies have evaluated the potential efficacy of traditional NSAIDs, such as diclofenac and ibuprofen (as nonselective COX-2 inhibitors) as well as ETD, celecoxib and piroxicam (as COX-2 selective inhibitor) for pain management. When a randomized clinical comparison was carried out between ETD and ibuprofen. Pain, swelling, and trismus after dental extraction operation were controlled effectively with ETD more than ibuprofen. ETD group experienced lighter pain and reduced trismus, because ETD inhibited the COX-2 enzyme which assumes a function in PG synthesis and metabolism in inflammation^59^. ETD demonstrated a significant selectivity of for the isozymes of prostaglandin G/H synthase (rhPGHS-2), it is more potent in chronic inflammatory conditions compared to acute cases^60^. ETD decreases the inflammatory macrophages outflow to the site of the chronic inflammation^61^. Besides, it is was reported that ETD diminish the synovial inflammation by down regulation of protein responsible for complement and coagulation cascade as well as platelets degranulation^62^.

## Methods

### Material and animals

Etodolac (ETD) was kindly donated by the European Egyptian Pharmaceuticals, Egypt. Phosal® 53 MCT, Lipoid S75 (hydrogenated soy bean phospholipids, ~ 69%) were gifted by Lipoid Co. (Ludwigshafen, Germany). Cholesterol, phenol, and rhodamine B were purchased from Oxford Lab Chen, India. Tween80, sodium deoxycholate (SDC), Span 20, poloxamer 188 were purchased from sigma Aldrich, Japan. Sodium hyaluronate (HA) (1.5–1.8 million Da) was from Shaanxi Green Bio-Engineering Co., Ltd (China). Formalin (37%) was purchased from Loba Chemie Pvt. Ltd, India. Voltaren emulgel® brand product of (Novartis Co., Swiss) was purchased from a local pharmacy. All other chemicals were of analytical grade and used without purification.

### Animals

For all subsequent experiments, female rats (250 ± 20 g) were obtained from the Faculty of Pharmacy Animal House (Alexandria, Egypt). They were fed with standard rodent food pellets and water ab libitum and housed in plastic cages in temperature-controlled rooms. Approval of ethical committee was obtained, and animals were handled according to the ethical guidelines of Alexandria University, AlexU-IACUC (Institutional Animal Care and Use Committee, a member of ICLAS) with an approval number (Au: AU/06,201,939). The study complies with the ARRIVE guidelines and the American Veterinary Medical Association (AVMA) Guidelines for the Euthanasia of Animals (2020).

### Preparation of ETD–PHs

The preliminary screening and optimization of different process and formulation variables were elaborated. Two methods were compared for the development of ETD–PHs: thin film hydration (FH) and ethanol injection (EI). For both techniques, Phosal® was used as the phosphatidylcholine-containing oily phase.

#### Film-hydration method (FH)

Phosalosomes (PHs) were prepared as previously reported by NK Abo Aasy^63^. In particular, Phosal®, cholesterol (0.07% w/v), and tween 80 were co-dissolved in chloroform in a 500-mL round-bottomed flask. The ETD was dissolved in methanol and added to the former mixture. The mixture was then evaporated under reduced pressure using a rotary evaporator (Rotavapor, type R110, Buchi, Flawil, Switzerland) at 40 °C and forms a thin dry film. The dried film was rehydrated with a phosphate buffer (PB, pH 5.5) and thereafter shaken until it was fully resuspended. The formulations were homogenized using a high-speed homogenizer (ULTRA-TURAX T25, IKA Labortechnik, Germany) and thereafter sonicated (Julabo sonicator, model USR3, JulaboLabortechnik, Ceelbach, Germany).

#### Ethanol injection–sonication (EI) method

Aliquot, approximately 1 mL of the lipid mixture (Phosal®, CH, and tween 80) in ethanol, was injected at room temperature (RT) into a magnetically stirred water (Ika, Labortechnik, Germany). The produced dispersion was diluted using double-filtered water (1:50) and vigorously shaken for complete ethanol removal; thereafter, it is sonicated for 15 min^48^.

### Optimization of tailored PHs

In the on-going study, a preliminary screening and optimization of different process and formulation variables were elaborated. The determining factors for optimization were the PHs micromeritics: particle size (PS), zeta potential (ζP) along with its polydispersity index (PDI). Data analysis was performed using one-way analysis of variance test (ANOVA). The Two-tailed t-test was used to determine the level of significance among paired tested formulations. The difference was considered statistically significant at a level of *P* value < 0.05. The level of significance among paired tested formulations was tested using Two-tailed t-test.

#### Optimization of process variables

Different process variables, such as the preparation method (either FH or EI), size uniformity techniques including homogenization speed (4200, 6700, and 12 000 rpm), homogenization time (10, 15, and 30 min), and sonication time (5, 15, and 30 min), use of probe sonicator operated for 5 min (8 s on and 1 s off at 50 W and 50% frequency) were systematically investigated.

#### Optimization of formulation variables

Phosalosomal formulations were optimized, dispersion with no external surfactant was compared with those containing 10 mg (%w/w) of either Tween 80, Span 20, a mixture of Tween 80 and Poloxamer 188 (1:1 w/w), or a mixture of Tween 80, Poloxamer 188, and SDC (1:1:0.25w/w/w). Rehydration media of either water, Phosphate buffer saline (PBS, pH 7.4) and PB (pH 5.5) were tried. Phosal® concentration (1.5, 3, and 5%w/v), and ETD concentration (0.02, 0.04, 0.06, 0.08%w/v and scaling up to 1 g %w/v) were examined.

### Physicochemical characterization of ETD-PHs

Prepared formulations were characterized according to Particle size, PDI, ζP, Entrapment Efficiency (EE%), and drug content. Moreover, morphological elucidation using Transmission and scanning electron microscopy (TEM and SEM) was performed. Fourier Transform Infrared studies (FT-IR) spectra of ETD, Phosal®, ETD- Phosal® physical mixture, blank PHs dispersion and ETD-PHs dispersion and Differential scanning calorimetry (DSC) thermograms of pure ETD, mannitol powder and lyophilized ETD-PHs dispersion were examined. Detailed of characterization experiments were kept in details in the supplementary information.

### Hyaluronic acid (HA) gel formulation and characterization

#### Hyaluronic acid (HA) gel formulation

1% w/w ETD loaded phosalosomal gel (ETD-PHs-HA) was formulated using sodium hyaluronate (HA) as a gelling agent. 1% HA gel was prepared by dispersing the exact amount of HA in ETD-PHs dispersion and stirring at 80 rpm using magnetic stirrer till complete dissolution and then left for 24 h till complete swelling. Prepared ETD-PHs-HA gel was compared with those gels prepared by dissolving 1% HA in either distilled water (blank) and ETD buffer solution (ETD-HA).

#### Hyaluronic acid (HA) gel characterization

Prepared gels were checked for clarity by visual inspection. The pH values of the prepared gel formulations were checked using digital pH meter (Adwa 1030 digital pH /mV meter Romania, Europe) at constant temperature. Rheological behavior of all formulated gels at RT was studied using Brookfield digital Viscometer (DV 2T, Engineering Laboratories, INC., USA) fitted with S-40 Spindle at 15, 100, 150, 200 rpm and shear rate range from112.5 to 1500 s^−1^, keeping the torque value ranging from 33.8 to 80%. The average viscosity of the gel formulations was calculated over 1 min. The spreadability of the prepared HA gel formulations was determined 48 h after preparation, by measuring the spreading diameter of 1 g of the gel between two glass plates in a pre-circled area. The mass of the upper plate was increased from 100, 250 to 500g for 30 s each. The increase in the diameter of the circled area due to gel spreading after each addition was recorded. The procedure was repeated 3 times and the average was calculated. The spreadability factor was measured.

1 $$ {\text{Sf}} = {\text{A}}/{\text{W}} $$

where Sf (cm^2^ g^−1^) is the spreadability factor, defined as the ratio of the maximum spread area, (A) in cm^2^, after the addition of the weights to the total weight added (W) in g^42^.

### In vitro release assessment

The in-vitro release of ETD was carried out by dialysis bag method. Aliquots equivalent to 10 mg ETD from 0.04, 0.06 mg, 1% ETD-PHs dispersions, 1% ETD-PHs- HA gel were filled in the dialysis bag and compared with ETD suspension with the same strength prepared in PB (pH5.5) which is the external phase of the dispersions. The results were expressed as mean ± S.D. Data was presented as cumulative % ETD release against time in h and furtherly fitted to release models to figure out release model and mechanism. T_50_ was also used as a comparative tool between the release profiles. Detail of release experiment, and HPLC quantification assay of ETD were set in the supplementary information.

### Ex vivo permeation and retention study

#### Skin preparation

The full-thickness dorsal skin of female rats was dehaired prior to excision. The skin was cut into pieces suitable for placement into the Franz receptor cell. Thereafter, the pieces were soaked in the PBS (pH 7.4) for 30 min prior to the experiment.

#### Permeation test

The ex-vivo skin permeation of the ETD from the ETD suspension, ETD–PHs, and ETD–PHs-HA over 24 h through rat skin was assessed using a Franz cell assembly mounted in a glass beaker. The skin was mounted between the donor and acceptor compartments. Aliquots of 1 mL (equivalent to 10 mg ETD) of each of the tested formulations were placed over the donor compartments, and the receiver compartments were filled with 8 mL of PBS (pH 7.4). Then, the glass beakers were placed in a thermo-stated shaking water bath adjusted at 32 °C and 75 rpm without the application of any occlusive conditions. Each study was performed in triplicate. The entire release media were withdrawn from the receiver compartments through the side tube every hour for up to 8 h then after 24 h. At each time interval, a fresh PBS (pH 7.4) was used for compensation to maintain constant volume. The samples were filtered through 0.22-µm syringe filters and analyzed by the HPLC^64^.

#### Permeation data analysis

Data were presented as a plot of the cumulative amount of ETD permeated in (µg/cm^2^) versus time in (h) for each formulation. The drug permeation parameters were calculated according to Marwa et al.^65^ where, *J*_ss_ which is the drug permeation flux at steady state (in µg/cm^2^ h) was calculated from the slope of the linear portion of the plot. The permeability coefficient, Kp (in cm/h), was calculated using the following equation

2 $$ {\text{Kp}} = J_{{{\text{ss}}}} /{\text{C}}_{0} $$

where, C_0_ is the initial drug concentration in a 1 mL formula added to the donor cell. The diffusion coefficient ‘‘D’’ was calculated by multiplying the Kp with the skin thickness.

#### Retention study

At the end of the permeation study, the retained drug inside the skin was retrieved and quantified. The skin was removed from the Franz cell, cleaned with gauze, and meticulously washed with the PBS (pH 7.4) followed by distilled water. The permeation area of the skin was then excised and cut into small pieces. The drug contained therein was extracted with 10 mL of methanol for 8 h; thereafter, 10 mL of PBS (pH 7.4) was added, left for 24 h, and then sonicated for 20 min. The resulting solutions were measured by the HPLC, which measured the amount of drug retained in the skin expressed in (μg/cm^2^)^66^.

### Visualization of dermal distribution

To ascertain the penetration depth, strength of the formulation permeation, penetration pathway and dermal distribution, a fluorescence visualization experiment was performed. Etodolac, was replaced by a fluorescent-dye, Rhodamine B(RH). Rhodamine B(RH) was loaded into PHs-HA gel (RH-PHs-HA), PHs dispersion (RH-PHs) and dye solution at the same strength as ETD (1% w/v). The rats were shaved on their dorsal sides 24 h before starting the experiment. RH loaded formulations were applied topically to a 2 cm^2^ area of the shaved dorsal region of female rats (250 ± 20 g) and kept under dark conditions. After 6 h, the animals were sacrificed after anaesthesia with ketamine (70 mg/kg, I.P), and skin samples were excised. The collected skin samples were carefully washed with normal saline and water (three times each) to remove excess dye. Thereafter, the skin pieces were suspended in 10% v/v formalin in preparation for wax cubing; these were sliced into vertical cross-sections with a maximum thickness of 6 µm. These sections were observed under a florescent microscope (Olympus EX-41, equipped by Olympus U-RFL-T fluorescence lamp, Japan) and photographed; the fluorescence intensity was quantified using image J software (ij-154-win-java 8, https://imagej.net/ij/)^42^.

### Quantitative biodistribution assay

One of the merits of site-specific drug delivery systems is it can minimize the non-specific distribution of the drug to other vital organs (liver and kidney) and thus avoid potential toxicities (hepatotoxicity and nephrotoxicity, respectively). An integral and crucial fluorescent biodistribution study was elaborated to visualize the distribution of topically applied RH-PHs-HA gel versus the distribution of systemically administrated RH solution (intraperitoneal injection, I.P). The rats were randomly divided into two groups (each of three rats). Group I: the rats received a 3 mL IP of RH solution (20 mg/mL). In group II: the backs of the rats were shaved (2 cm^2^ area) 24 h prior to the study. The rats received a topical dose of RH-PHs-HA gel (100 µL, 20 mg/mL) on their shaved skin areas. After period of 8 h, animals were anesthetized with ketamine (70 mg/kg, I.P) and then sacrificed, and their vital organs of elimination (spleen, liver and kidney) and skins were collected, washed with saline (0.9% NaCl) solution. The tissues were fixed using the conventional procedure, and the prepared slides were examined for the quantitative analysis of the fluorescence intensity in each organ under Olympus EX-41 confocal laser microscope (LSCM, Leica TSC SPE II DMi8, Germany). The fluorescence intensity was measured using LasX software (https://www.leica-microsystems.com/products/microscope-software/p/leica-las-x-ls/) as average pixels per µm^2^ ± SD^67^.

### In vivo skin irritation test

#### Contact dermatitis experiment design

The topical application of the formulations on the skin may be attributed to certain types of skin irritation. To determine the degree of skin irritation, this test was performed on the back skin of female rats (250 ± 20 g). Their backs were shaved with a razor 24 h prior to the test. The rats were divided into four groups, each group constitutes six rats. The first group was treated with normal saline (negative control). The second group was topically treated with 100 µL, 5% phenol; it served as the positive control group. The third group was topically treated with 100 µL, 1% ETD–PHs–HA gel. For the last group, the ETD solution with the same strength was applied. All treatments were applied to the 2-cm^2^ area of the dorsal surface of rats for six consecutive days. To check for any acute allergic reactions (6 h after the first dose), three rats per group were sacrificed after I.P injection with ketamine (70 mg/kg), and skin samples were collected. The rest of the rats were observed daily for any erythema, edema, or scaring. On the fifteenth day, the remaining three rats per each group were sacrificed; and skin samples were collected to be tested for chronic allergic reaction^42^.

#### Histopathological examination

The excised skin samples were first washed three times with normal saline as part of the preparation for histological examination. Thereafter, the skin samples were dried and set in a 10% v/v formalin solution. The sections were dehydrated using alcohol, preserved in a paraffin block, cut into 6-µm slices; thereafter, these were stained with hematoxylin and eosin (H&E stains) and observed under an optical microscope (Olympus EX-41, equipped with Olympus DP20 camera, USA).

### In vivo anti-inflammatory and antinociception potential

#### Phenol induced ear edema model (Acute anti-inflammatory test)

The anti-inflammatory potential of the ETD-PHs-HA gel was evaluated by the edema induction method. Fifteen female rats (250 ± 20 g) were divided into five groups (each of three rats. Ear edema was induced in only four of these groups. In group I: no application was made (negative control). Group II was treated only with 20μL of the inflammatory agent; 5% w/v phenol, subcutaneously applied in outer side of the right ear and kept as a positive control. Group III was treated with ETD solution (100 µL), Group IV was treated with ETD-PHs-HA gel (100 µL). Group V was treated with an anti-inflammatory brand product for potency comparison.

After 30 min, Group III, IV, and V were treated with 20μL 5% w/v phenol as Group II. The animals were sacrificed four h after the administration of phenol. The ears were individually cut from the base and weighted on an analytical balance. The edema was measured as the difference between the weight of the right and left (non-inflamed) ears. The results were then expressed as the inflammation degree, which is the average increase in ear weight (in mg) ± SD. Moreover, the anti-inflammatory activity (% inhibition) in the treated groups was also calculated according to the following equation^68^.

3 $$ \% \,~{\text{Inhibition}}~ = ~\left[ {100 - ~\frac{{{\text{average~increase~in~ear~weight~of~positive~control~grpoup}} - {\text{average~increase~in~weight~of~treated~ear~grpoup}}}}{{{\text{average~increase~in~ear~weight~of~positive~control~grpoup}}}}} \right] $$

Subsequently, circular sections (7 mm in diameter) were cut and collected from both right and left ears. The excised skin samples were prepared for histological examination as previously described. Skin irritation was observed under an optical microscope, and ear thickness was measured with Olympus software (DP20-DRV (Ver.01.02,https://www.olympus-lifescience.com/en/support/downloads/dp20-dev_ver0102_device_drv_step/).

#### Formalin-induced nociception assay

Before this test, male rats weighing 250 ± 20 g were placed in an observation cage. The rats were divided into four groups (each of three rats): Group I was treated with ETD-PHs-HA gel (100 µL), Group II was treated with an anti-inflammatory brand product for potency comparison and Group III was treated with blank PHs-HA gel. Group IV: was treated with formalin only (positive control).

Nociception was induced by the subcutaneous injection of 5% v/v formalin into the hind paw of the rats (20 µL per paw). The treatments were topically applied on the dorsal surface of the rat paw (100 µL per paw) for 30 min prior to the administration of formalin to the aforementioned three groups. The formalin injection induced responses such as licking and/or biting the injected paw, in rats. Both behaviors reflected the degree of pain experienced by the animals. The total time (min) that each animal spent licking or biting its paw was recorded during the early and late phase of formalin-induced nociception with a hand-held stopwatch; these observations were considerably analyzed^68^.

### Stability study

The ETD-PHs dispersion was stored in a well-closed glass container for three months at 2–8 °C. The dispersion was then assessed for in vitro release profile, physical appearance, ζP, and particle size, before and after storage. The stability was assessed by visual inspection for drug precipitation or loss of consistency at the end the aforementioned period. Stability of the prepared gel formulations was also monitored visually after storing in sealed jar for one month.

### Statistical analysis

Data analysis for all previous studies was performed using one-way analysis of variance test (ANOVA). The Two-tailed t-test was used to determine the level of significance among paired tested formulations (DD solver Excel Add in). The difference was considered statistically significant at a level of *P* value < 0.05.

## Conclusions

The incorporation of ETD into PHs resulted in water-dispersible and stable Phosal®-based dispersions. Homogenization time and speed, sonication time, surfactant type, rehydration media and different size reduction methods were screened to obtain optimized ETD-loaded PHs with a significantly reduced size and high negative ζP. The optimized formula was composed of 1% w/v ETD, Phosal®, CH and Tween 80, using PB (pH = 5.5) as a rehydration media. The optimized homogenization time was 15 min at 4200 rpm followed by sonication for 30 min. Final colloidal dispersion was gelled using HA gel to control ETD in-vitro release and ensure good spreadability. ETD-PHs-HA gel was successfully facilitated the localization of ETD into the skin layers 10.59 folds more compared with free ETD. ETD-PHs-HA gel revealed remarkable skin tolerability upon histopathological examination after acute and chronic application on rat skin. After topical gel application, the dose distributed in the vital organs was significantly lower than I.P dose with significant an anti-oedemic and anti-nociception potential. The enhanced localization of PHs emphasized the potential of the system for the delivery of more lipophilic drugs. Meanwhile, open the door for the researchers to incorporate ETD in novel nanocarriers for improved effect against other skin conditions and systemic application.

## Supplementary Information

Supplementary Information.

## Data Availability

All data generated and analysed during the study are provided within the manuscript or supplementary information files.

## References

[1] Wang, H. *et al.* Update on nanoparticle-based drug delivery system for anti-inflammatory treatment. *Front. Bioeng. Biotechnol.* **9**, 630352. 10.3389/fbioe.2021.630352 (2021). 33681167 10.3389/fbioe.2021.630352 PMC7925417

[2] Shao, A. *et al.* Hydrogen-rich saline attenuated subarachnoid hemorrhage-induced early brain injury in rats by suppressing inflammatory response: possible involvement of NF-κB pathway and NLRP3 inflammasome. *Mol. Neurobiol.* **53**, 3462–3476 (2016). 26091790 10.1007/s12035-015-9242-y

[3] Shahraki, O., Shayganpour, M., Hashemzaei, M. & Daneshmand, S. Solid lipid nanoparticles (SLNs), the potential novel vehicle for enhanced in vivo efficacy of hesperidin as an anti-inflammatory agent. *Bioorganic Chem.* **131**, 106333 (2023). 10.1016/j.bioorg.2022.106333 36587504

[4] Kazim, T., Tariq, A., Usman, M., Ayoob, M. F. & Khan, A. Chitosan hydrogel for topical delivery of ebastine loaded solid lipid nanoparticles for alleviation of allergic contact dermatitis. *RSC Adv.* **11**, 37413–37425 (2021). 35496417 10.1039/d1ra06283b PMC9043795

[5] Mark, B. J. & Slavin, R. G. Allergic contact dermatitis. *Med. Clin.* **90**, 169–185 (2006). 10.1016/j.mcna.2005.08.008 16310529

[6] Kimber, I., Basketter, D. A., Gerberick, G. F. & Dearman, R. J. Allergic contact dermatitis. *Int. Immunopharmacol.* **2**, 201–211 (2002). 11811925 10.1016/s1567-5769(01)00173-4

[7] Kasting, G. B., Barai, N. D., Wang, T. F. & Nitsche, J. M. Mobility of water in human stratum corneum. *J. Pharm. Sci.* **92**, 2326–2340 (2003). 14603517 10.1002/jps.10483

[8] Knorr, F. *et al.* Follicular transport route–research progress and future perspectives. *Eur. J. Pharm. Biopharm.* **71**, 173–180 (2009). 19041720 10.1016/j.ejpb.2008.11.001

[9] Trauer, S. *et al.* Influence of massage and occlusion on the ex vivo skin penetration of rigid liposomes and invasomes. *Eur. J. Pharm. Biopharm.: Off. J. Arbeitsgemeinschaft fur Pharmazeutische Verfahrenstechnik eV* **86**, 301–306 (2014). 10.1016/j.ejpb.2013.11.004 24252713

[10] Madawi, E. A. *et al.* Polymeric nanoparticles as tunable nanocarriers for targeted delivery of drugs to skin tissues for treatment of topical skin diseases. *Pharmaceutics* **15**, 657 (2023). 36839979 10.3390/pharmaceutics15020657 PMC9964857

[11] Akhtar, N., Verma, A. & Pathak, K. Exploring preclinical and clinical effectiveness of nanoformulations in the treatment of atopic dermatitis: Safety aspects and patent reviews. *Bull. Fac. Pharm., Cairo Univ.* **55**, 1–10 (2017).

[12] Jones, R. A. Etodolac (Lodine®): Profile of an established selective COX-2 inhibitor. *Inflammopharmacology* **9**, 63–70 (2001).

[13] Brocks, D. R. & Jamali, F. Etodolac clinical pharmacokinetics. *Clin. Pharmacokinet.* **26**, 259–274 (1994). 8013160 10.2165/00003088-199426040-00003

[14] Barakat, N. S. Etodolac-liquid-filled dispersion into hard gelatin capsules: an approach to improve dissolution and stability of etodolac formulation. *Drug Dev. Ind. Pharm.* **32**, 865–876. 10.1080/03639040500534192 (2006). 16908424 10.1080/03639040500534192

[15] Tas, C., Ozkan, Y., Okyar, A. & Savaser, A. In vitroandex vivopermeation studies of etodolac from hydrophilic gels and effect of terpenes as enhancers. *Drug Deliv.* **14**, 453–459. 10.1080/10717540701603746 (2008). 10.1080/10717540701603746 17994363

[16] Goindi, S., Kaur, R. & Kaur, R. An ionic liquid-in-water microemulsion as a potential carrier for topical delivery of poorly water soluble drug: Development, ex-vivo and in-vivo evaluation. *Int. J. Pharm.* **495**, 913–923. 10.1016/j.ijpharm.2015.09.066 (2015). 26456294 10.1016/j.ijpharm.2015.09.066

[17] Jain, S., Patel, N., Shah, M. K., Khatri, P. & Vora, N. Recent advances in lipid-based vesicles and particulate carriers for topical and transdermal application. *J. Pharm. Sci.* **106**, 423–445. 10.1016/j.xphs.2016.10.001 (2017). 27865609 10.1016/j.xphs.2016.10.001

[18] Manca, M. L. *et al.* Development of curcumin loaded sodium hyaluronate immobilized vesicles (hyalurosomes) and their potential on skin inflammation and wound restoring. *Biomaterials* **71**, 100–109. 10.1016/j.biomaterials.2015.08.034 (2015). 26321058 10.1016/j.biomaterials.2015.08.034

[19] Wang, W. X., Feng, S. S. & Zheng, C. H. A comparison between conventional liposome and drug-cyclodextrin complex in liposome system. *Int. J. Pharm.* **513**, 387–392. 10.1016/j.ijpharm.2016.09.043 (2016). 27640244 10.1016/j.ijpharm.2016.09.043

[20] Zhao, W. *et al.* Caprylic triglyceride as a novel therapeutic approach to effectively improve the performance and attenuate the symptoms due to the motor neuron loss in ALS disease. *PloS One* **7**, e49191. 10.1371/journal.pone.0049191 (2012). 23145119 10.1371/journal.pone.0049191 PMC3492315

[21] Shah, P. P., Desai, P. R., Patel, A. R. & Singh, M. S. Skin permeating nanogel for the cutaneous co-delivery of two anti-inflammatory drugs. *Biomaterials* **33**, 1607–1617. 10.1016/j.biomaterials.2011.11.011 (2012). 22118820 10.1016/j.biomaterials.2011.11.011 PMC3242008

[22] Zhang, Y. *et al.* Ascorbyl palmitate/d-alpha-tocopheryl polyethylene glycol 1000 succinate monoester mixed micelles for prolonged circulation and targeted delivery of compound K for antilung cancer therapy in vitro and in vivo. *Int. J. Nanomed.* **12**, 605–614. 10.2147/IJN.S119226 (2017). 10.2147/IJN.S119226 PMC5248941 28144142

[23] Moldovan, M. *et al.* Formulation and evaluation of a water-in-oil cream containing herbal active ingredients and ferulic acid. *Clujul Med.* **90**, 212–219 (2017). 28559707 10.15386/cjmed-668 PMC5433575

[24] Gallarate, M., Chirio, D., Trotta, M. & Eugenia Carlotti, M. Deformable liposomes as topical formulations containing α-tocopherol. *J. Dispers. Sci. Technol.* **27**, 703–713 (2006).

[25] Allam, A. N., Komeil, I. A., Fouda, M. A. & Abdallah, O. Y. Preparation, characterization and in vivo evaluation of curcumin self-nano phospholipid dispersion as an approach to enhance oral bioavailability. *Int. J. Pharm.* **489**, 117–123. 10.1016/j.ijpharm.2015.04.067 (2015). 25936626 10.1016/j.ijpharm.2015.04.067

[26] Shanmugam, S. *et al.* Enhanced bioavailability and retinal accumulation of lutein from self-emulsifying phospholipid suspension (SEPS). *Int. J. Pharm.* **412**, 99–105 (2011). 21540096 10.1016/j.ijpharm.2011.04.015

[27] Shehata, E. M. M., Elnaggar, Y. S. R., Galal, S. & Abdallah, O. Y. Self-emulsifying phospholipid pre-concentrates (SEPPs) for improved oral delivery of the anti-cancer genistein: Development, appraisal and ex-vivo intestinal permeation. *Int. J. Pharm.* **511**, 745–756. 10.1016/j.ijpharm.2016.07.078 (2016). 27492016 10.1016/j.ijpharm.2016.07.078

[28] Puré, E. & Cuff, C. A. A crucial role for CD44 in inflammation. *Trends Mol. Med.* **7**, 213–221 (2001). 11325633 10.1016/s1471-4914(01)01963-3

[29] Teder, P. *et al.* Resolution of lung inflammation by CD44. *Science* **296**, 155–158. 10.1126/science.1069659 (2002). 11935029 10.1126/science.1069659

[30] Wang, D. *et al.* The eradication of breast cancer cells and stem cells by 8-hydroxyquinoline-loaded hyaluronan modified mesoporous silica nanoparticle-supported lipid bilayers containing docetaxel. *Biomaterials* **34**, 7662–7673. 10.1016/j.biomaterials.2013.06.042 (2013). 23859657 10.1016/j.biomaterials.2013.06.042

[31] Tripodo, G. *et al.* Hyaluronic acid and its derivatives in drug delivery and imaging: Recent advances and challenges. *Eur. J. Pharm. Biopharm.: Off. J. Arbeitsgemeinschaft fur Pharmazeutische Verfahrenstechnik eV* **97**, 400–416. 10.1016/j.ejpb.2015.03.032 (2015). 10.1016/j.ejpb.2015.03.032 26614559

[32] Jaafar-Maalej, C., Diab, R., Andrieu, V., Elaissari, A. & Fessi, H. Ethanol injection method for hydrophilic and lipophilic drug-loaded liposome preparation. *J. Liposome Res.* **20**(3), 228–243 (2010). 19899957 10.3109/08982100903347923

[33] Gupta, S. & Vyas, S. P. Development and characterization of amphotericin B bearing emulsomes for passive and active macrophage targeting. *J. Drug Target.* **15**, 206–217. 10.1080/10611860701195395 (2007). 17454358 10.1080/10611860701195395

[34] Shaker, S., Gardouh, A. R. & Ghorab, M. M. Factors affecting liposomes particle size prepared by ethanol injection method. *Res. Pharm. Sci.* **12**(5), 346–352 (2017). 28974972 10.4103/1735-5362.213979 PMC5615864

[35] Han, F., Li, S., Yin, R., Liu, H. & Xu, L. Effect of surfactants on the formation and characterization of a new type of colloidal drug delivery system: Nanostructured lipid carriers. *Colloids Surf. A: Physicochem. Eng. Asp.* **315**, 210–216. 10.1016/j.colsurfa.2007.08.005 (2008).

[36] Alam, M. I., Paget, T. & Elkordy, A. A. Formulation and advantages of furazolidone in liposomal drug delivery systems. *Eur. J. Pharm. Sci.: Off. J. Eur. Fed. Pharm. Sci.* **84**, 139–145. 10.1016/j.ejps.2016.01.017 (2016). 10.1016/j.ejps.2016.01.017 26796143

[37] Shah, K. P., Gumbhir-Shah, K. & Brittain, H. G. *Etodolac profile* Vol. 29, 109–114 (Elsiever science, Amsterdam, 2002).

[38] Shah, K. P., Gumbhir-Shah, K., & Brittain, H. G. Etodolac, in *Analytical profiles of drug substances and excipients*, Vol. 29, pp. 105-147. (Academic Press, 2002).

[39] Barreneche, C., Gil, A., Sheth, F., Fernández, A. I. & Cabeza, L. F. Effect of d-mannitol polymorphism in its thermal energy storage capacity when it is used as PCM. *Solar Energy* **94**, 344–351 (2013).

[40] Sallam, M. A. & Boscá, M. T. M. Mechanistic analysis of human skin distribution and follicular targeting of adapalene loaded biodegradable nanospheres with an insight into hydrogel matrix influence, in-vitro skin irritation and in-vivo tolerability. *J. Pharm. Sci.* **106**, 3140–3149 (2017). 28603018 10.1016/j.xphs.2017.05.038

[41] Im-Emsap W. S. J., Paeratakul O. *Modern Pharmaceutics* 4th edn, 243 (Marcel Dekker, Inc, 2002).

[42] Jain, A., Doppalapudi, S., Domb, A. J. & Khan, W. Tacrolimus and curcumin co-loaded liposphere gel: Synergistic combination towards management of psoriasis. *J. Controll. Release* **243**, 132–145. 10.1016/j.jconrel.2016.10.004 (2016). 10.1016/j.jconrel.2016.10.004 27725194

[43] Koizuka, S. *et al.* Oral etodolac, a COX-2 inhibitor, reduces postoperative pain immediately after fast-track cardiac surgery. *J. Anesthesia* **18**, 9–13. 10.1007/s00540-003-0215-3 (2004). 10.1007/s00540-003-0215-3 14991469

[44] Pireddu, R. *et al.* Diclofenac acid nanocrystals as an effective strategy to reduce in vivo skin inflammation by improving dermal drug bioavailability. *Colloids Surf. B, Biointerfaces* **143**, 64–70. 10.1016/j.colsurfb.2016.03.026 (2016). 26998867 10.1016/j.colsurfb.2016.03.026

[45] Carafa, M. *et al.* Ammonium glycyrrhizinate-loaded niosomes as a potential nanotherapeutic system for anti-inflammatory activity in murine models. *Int. J. Nanomed.*10.2147/ijn.s55066 (2014). 10.2147/IJN.S55066 PMC3908944 24493924

[46] Ucisik, M. H., Küpcü, S., Schuster, B. & Sleytr, U. B. Characterization of CurcuEmulsomes: Nanoformulation for enhanced solubility and delivery of curcumin. *J. Nanobiotechnol.* **11**, 37 (2013). 10.1186/1477-3155-11-37 PMC4029586 24314310

[47] Gill, B., Singh, J., Sharma, V. & Kumar, S. H. Emulsomes: An emerging vesicular drug delivery system. *Asian J. Pharm.* **6**, 87 (2012).

[48] Gupta, R., Gupta, M., Mangal, S., Agrawal, U. & Vyas, S. P. Capsaicin-loaded vesicular systems designed for enhancing localized delivery for psoriasis therapy. *Artif. Cells Nanomed. Biotechnol.* **44**, 825–834. 10.3109/21691401.2014.984301 (2016). 25465045 10.3109/21691401.2014.984301

[49] Pal, A., Gupta, S., Jaiswal, A., Dube, A. & Vyas, S. P. Development and evaluation of tripalmitin emulsomes for the treatment of experimental visceral leishmaniasis. *J. Liposome Res.* **22**, 62–71. 10.3109/08982104.2011.592495 (2011). 21740098 10.3109/08982104.2011.592495

[50] Verma, D. D., Verma, S., Blume, G. & Fahr, A. Particle size of liposomes influences dermal delivery of substances into skin. *Int. J. Pharm.* **258**, 141–151 (2003). 12753761 10.1016/s0378-5173(03)00183-2

[51] Mohyeldin, S. M., Mehanna, M. M. & Elgindy, N. A. Superiority of liquid crystalline cubic nanocarriers as hormonal transdermal vehicle: Comparative human skin permeation-supported evidence. *Expert Opin. Drug Deliv.* **13**, 1049–1064. 10.1080/17425247.2016.1182490 (2016). 27167758 10.1080/17425247.2016.1182490

[52] Nakach, M. *et al.* Assessment of formulation robustness for nano-crystalline suspensions using failure mode analysis or derisking approach. *Int. J. Pharm.* **506**, 320–331. 10.1016/j.ijpharm.2016.04.043 (2016). 27102992 10.1016/j.ijpharm.2016.04.043

[53] Shah, S. M., Konda, N. R., Palle, P. G., Nuvvula, K. & Gannimetta, A. Formulation and characterization of doxorubicin hydrochloride liposomes by double emulsion method. *Int. Res. J. Pharm.* **4**, 197–201. 10.7897/2230-8407.04439 (2016).

[54] Han, F., Li, S., Yin, R., Liu, H. & Xu, L. Effect of surfactants on the formation and characterization of a new type of colloidal drug delivery system: Nanostructured lipid carriers. *Colloids Surf. A: Physicochem. Eng. Asp.* **315**, 210–216. 10.1016/j.colsurfa.2007.08.005 (2008).

[55] Lúcio, M., Ferreira, H., Lima, J. L. F. C. & Reis, S. Use of liposomes as membrane models to evaluate the contribution of drug–membrane interactions to antioxidant properties of etodolac. *Redox Rep.* **13**, 225–236. 10.1179/135100008x308939 (2013). 10.1179/135100008X308939 18796242

[56] du Plessis, J., Egbaria, K., Ramachandran, C. & Weiner, N. Topical delivery of liposomally encapsulated gamma-interferon. *Antivir. Res.* **18**, 259–265 (1992). 1416907 10.1016/0166-3542(92)90059-e

[57] Li, N., Peng, L.-H., Chen, X., Nakagawa, S. & Gao, J.-Q. Effective transcutaneous immunization by antigen-loaded flexible liposome in vivo. *Int. J. Nanomed.* **6**, 3241 (2011). 10.2147/IJN.S26152 PMC3252672 22228992

[58] Ortiz, M. I. *et al.* Isolation, identification and molecular docking as cyclooxygenase (COX) inhibitors of the main constituents of *Matricaria chamomilla* L. extract and its synergistic interaction with diclofenac on nociception and gastric damage in rats. *Biomed. Pharm. Biomed. Pharm.* **78**, 248–256. 10.1016/j.biopha.2016.01.029 (2016). 10.1016/j.biopha.2016.01.029 26898449

[59] Mutlu, V. & Ince, I. Preemptive intravenous ibuprofen application reduces pain and opioid consumption following thyroid surgery. *Am. J. Otolaryngol.* **40**, 70–73. 10.1016/j.amjoto.2018.10.008 (2019). 30472123 10.1016/j.amjoto.2018.10.008

[60] Glaser, K. *et al.* Etodolac selectively inhibits human prostaglandin G/H synthase 2 (PGHS-2) versus human PGHS-1. *Eur. J. Pharmacol.* **281**, 107–111 (1995). 8566109 10.1016/0014-2999(95)00302-2

[61] Gervais, F., Martel, R. R. & Skamene, E. The effect of the non-steroidal anti-inflammatory drug etodolac on macrophage migration in vitro and in vivo. *J. Immunopharmacol.* **6**, 205–214 (1984). 6238099 10.3109/08923978409019461

[62] Feng, Q. *et al.* Etodolac improves collagen induced rheumatoid arthritis in rats by inhibiting synovial inflammation, fibrosis and hyperplasia. *Mol. Biomed.* **2**, 33 (2021). 35006449 10.1186/s43556-021-00052-1 PMC8607370

[63] Abo Aasy, N. K. *et al.* A comparative study: the prospective influence of nanovectors in leveraging the chemopreventive potential of COX-2 inhibitors against skin cancer. *Int. J. Nanomed.* **14**, 7561–7581 (2019). 10.2147/IJN.S218905 PMC6756578 31571864

[64] Zaid Alkilani, A., Nimrawi, S., Al-Nemrawi, N. K. & Nasereddin, J. Microneedle-assisted transdermal delivery of amlodipine besylate loaded nanoparticles. *Drug Dev. Ind. Pharm.* **48**, 322–332 (2022). 35950766 10.1080/03639045.2022.2112694

[65] Sallam, M. A., Motawaa, A. M. & Mortada, S. M. An insight on human skin penetration of diflunisal: Lipogel versus hydrogel microemulsion. *Drug Dev. Ind. Pharm.* **41**, 141–147. 10.3109/03639045.2013.850711 (2015). 24171693 10.3109/03639045.2013.850711

[66] Yokota, J. & Kyotani, S. Influence of nanoparticle size on the skin penetration, skin retention and anti-inflammatory activity of non-steroidal anti-inflammatory drugs. *J. Chin. Med. Assoc.* **81**, 511–519 (2018). 29555445 10.1016/j.jcma.2018.01.008

[67] Gupta, V., Dhote, V., Paul, B. N. & Trivedi, P. Development of novel topical drug delivery system containing cisplatin and imiquimod for dual therapy in cutaneous epithelial malignancy. *J. Liposome Res.* **24**, 150–162. 10.3109/08982104.2013.865216 (2014). 24328725 10.3109/08982104.2013.865216

[68] Limón, D. *et al.* Novel nanostructured supramolecular hydrogels for the topical delivery of anionic drugs. *Eur. J. Pharm. Biopharm.: Off. J. Arbeitsgemeinschaft fur Pharmazeutische Verfahrenstechnik eV* **96**, 421–436. 10.1016/j.ejpb.2015.09.007 (2015). 10.1016/j.ejpb.2015.09.007 26409201

