# Supplementary material for: Follicular mediated etodolac phosalosomal gel for contact dermatitis alleviation, insights from optimization to in-vivo appraisal
Source: Sci Rep. 2024 Sep 18;14:21744. doi: 10.1038/s41598-024-71456-6 (PMC11408589; doi:10.1038/s41598-024-71456-6)
Supplement: Supplementary file 1 — Supplementary Information. [file 41598_2024_71456_MOESM1_ESM.pdf]

Supporting Information (SI)

Follicular Mediated Etodolac Phosalosomal Gel for Contact Dermatitis Alleviation, insights from Optimization to In-vivo Appraisal

Noha Khalifa Abo Aasy[1*], Doaa Ragab[1], Marwa Ahmed Sallam[1], and Kadria A. Elkhodairy[1]

[1] Department of Industrial Pharmacy, Faculty of Pharmacy, Alexandria University, Alexandria, Egypt.

***Corresponding Author: Noha Khalifa Abo Aasy**,

Department of Industrial Pharmacy, Faculty of Pharmacy, Alexandria University, 1 Khartoum Square, Azarita, Post Office, P.O. Box 21521, Alexandria, Egypt.

Tel: 00201227101058

Email: noha.abouassy@alexu.edu.eg

drnohakhalifa@yahoo.com

***ORCID**: 0000-0003-4348-0318*

1. Solubility Studies

As our first crucial mission is to improve ETD solubility by encapsulating in phosalosomes, solubility of pure ETD and ETD loaded phosalosomes in distilled water were measured. Moreover, as pre-formulation step, solubility of ETD at two different pH values (pH 7.7 and 5.5) was determined using a thermo-stated water bath (Staurt, SBS40, Staffordshire, Uk) at 37 °C and 100 rpm. ETD solutions were left for equilibration (24 h) and then filtered using PTFE syringe filters (0.22 µm). The concentration of ETD in the filtrate solution was determined using UV spectrophotometric method at 278 nm (T80 UV/VIS spectrometer, PG Instruments Ltd, United kingdom). As it difficult to assess ETD solubility by previous method, the solubility of ETD in phosal was assessed by successive addition of known quantities of ETD to a known volume of phosal, maintained at 37 °C till ETD precipitation. The total amount of ETD added was calculated. The solubility experiment was repeated in triplicate and the results were represented as mean value (mg/ml) ± standard deviation (SD).(1)

2. Physical characterization of ETD-PHs

2.1. Particle size, PDI and ζ P

Particle size of phosalosomal dispersions were analyzed by dynamic light scattering technique using Zetasizer (Malvern Instrument NanoZS, UK). ζ P values were calculated from the mean electrophoretic mobility obtained using the same instrument in a universal folded capillary cell, with embedded platinum electrodes. All samples were diluted in purified water and measured in three replicates and results were expressed as the mean value ± standard deviation (SD).

2.2. Drug Content and Entrapment Efficiency

The actual Drug content of ETD in phosalosomes was determined spectrophotometrically. A fixed volume of the dispersion (1 ml) was dissolved in methanol and diluted with PBS (pH7.4). The resultant solution was measured spectrophotometrically at λmax 278 nm against ETD-free phosalosomal solution diluted in the same manner as a blank.

With the aid of ultrafiltration process, the encapsulation efficiency (EE) of ETD-loaded phosalosomes could be calculated by separating non-entrapped drug. 1 ml aliquot of ETD dispersion was transferred into the upper chamber of a centrifuge tube fitted with an ultrafilter (Vivaspin Sartorius, MWCO 10 kDa) and centrifuged at 4000 rpm at 4°C (Sigma Laboratory Refrigerated Centrifuge, Model 3K-30, Germany) for 4 h. The aqueous filtrate was analyzed spectrophotometrically for quantifying the amount of ETD after appropriate dilution. Then, the amount entrapped was calculated indirectly by subtracting from the total drug used in the formulation. The percentage of EE was calculated as follows:

$$EE\% = 100 \times \left[M_i - M_f \big/ M_i \right] \qquad \text{Eq. 1}$$

where Mi is the mass of theoretical initial ETD, Mf is the mass of free ETD in the supernatant fluid and Mi is the mass of the phosalosomal dispersion. Each experiment was repeated three times and the values were expressed as mean ± S.D. (2)

2.3. Transmission and scanning electron microscopy (TEM, SEM)

In order to figure out and probe the microstructure of formulated vehicles, TEM (Joel, JEM-100 CX electron microscope) was used. Blank PHs prepared in the two buffers solutions pH 7.4 and 5.5 and applying the two methods (FH and EI) were photographed by (Joel, JEM-100 CX electron microscope). After proper sample dilution with distilled water, 1-2 sample drops were loaded to copper grid, left to dry and then photographed at different magnification powers using transmission electron microscope.

Scanning electron microscopy SEM (JOEL-JSM-6510, Japan) was adopted to examine the ability of PHs to maintain its (shape, size and surface structure) after lyophilization. First, phosalosomal dispersion was mixed with 5% mannitol, frozen at -80ºC for 24 hr and then freeze dried using Cryodos-50 lyophilizer (Telstar Cryodos, Terrassa, Spain). Subsequently, Sample were fixed on metal stubs using double-sided adhesive tape then coated with approximately 10-20 nm gold film for 20 s under vacuum using a sputter coater and then examined. Scan were performed at an acceleration voltage of 20 KV.

2.4. Fourier Transform Infrared studies (FT-IR)

FT-IR spectra of ETD powder, Phosal, ETD/Phosal physical mixture, blank PHs dispersion and ETD-PHs dispersion were obtained using Perkin-Elmer spectrum RXIFT-IR system, (Perkin-Elmer instruments, USA) to detect any drug/excipient interaction. The scanning range was 400 to 4000 cm-1 and the resolution was 1 cm-1.

2.5. Differential scanning calorimetry (DSC)

The thermal analysis of pure drug (ETD) powder, mannitol powder and lyophilized phosalosomal dispersion were performed using differential scanning calorimetry. 5mg of each sample was accurately weighed, placed into aluminium pans and then hermetically sealed with aluminium lids. The thermograms of the samples were obtained at scanning rate of 10 ºC/min conducted over a temperature range of 30 - 400 ºC.

3. In-Vitro drug release assay

3.1. Dialysis bag assay

In-vitro diffusion of ETD is carried out by dialysis bag method. Aliquots equivalent to 10 mg ETD from 40mg, 60 mg, 1% ETD-PHs dispersions, 1% ETD-PHs- HA gel were filled in the dialysis bag and compared with ETD suspension with the same strength prepared in PB (pH5.5) which is the external phase of the dispersions. Formulations were filled in the dialysis bag (Visking 36/32, 28 mm, MWCO 12,000–14,000; Serva, Heidelberg, Germany). The dialysis bag was maintained in a 250 ml glass beaker containing 100 ml of PB (pH 5.5) placed in a shaking thermo-stated water

bath at 32 °C and 75 rpm. 2 ml samples were taken at predetermined intervals and after 24 h, followed by compensation with fresh medium. After 24 h, the remaining formula in the dialysis bag was solubilized to determine unreleased ETD. All samples were analysed by HPLC method. Also, the diffusion of the 1% loaded phosalosomal loaded HA gel was performed in PBS (pH7.4) and PB (pH5.5) to mimic the inflammatory skin acidic conditions and to spot any change in the release profile. The results were expressed as mean ± S.D. Data was presented as % ETD release against time in h. $T_{50}$was used as a comparative tool be Tween the diffusion profiles.

3.2. HPLC qualification method

A simple sensitive and stability indicating HPLC method used by N. S. Barakat (3), for ETD content analysis was used with slight modification. The HPLC instrument (Perkin Elmer series 200; PerkinElmer Inc) equipped with UV detector. The column used was C18 (150 mm × 4.6 mm, 5 μ particle size). The mobile phase consisted of acetonitrile: potassium dihydrogen ortho- phosphate (0.04 M, pH 6.0) in a ratio of (35:65) at a flow rate 1 ml/min. Analysis was carried out at a wavelength of 278 nm. A calibration curve (peak area vs. drug concentration) was constructed by running the working standard solutions in the mobile phase. Stock standard solutions of ETD in PBS (pH7.4) were prepared to give a final concentration of 30 μg/ml. Standard solutions of ETD over the range 3-30 μg/ml were prepared by subsequent dilution (R^2=0.9958).

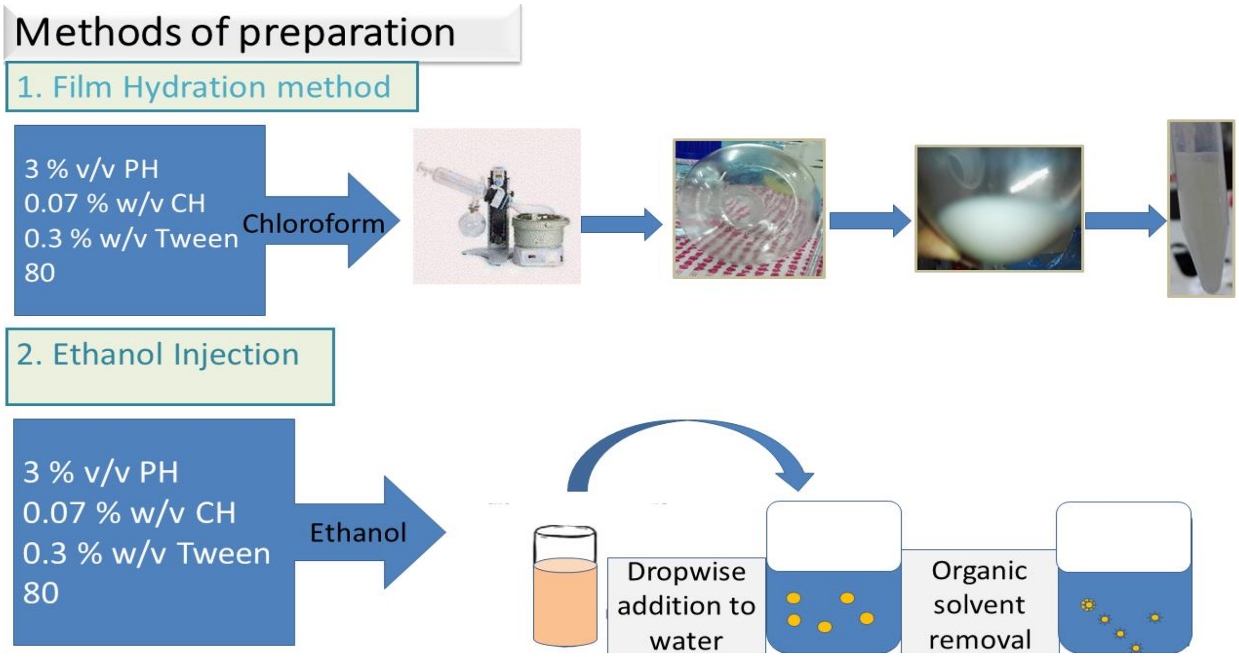

S1: Diagram of Film Hydration and Ethanol Injection methods used in Phosalosomes Preparation

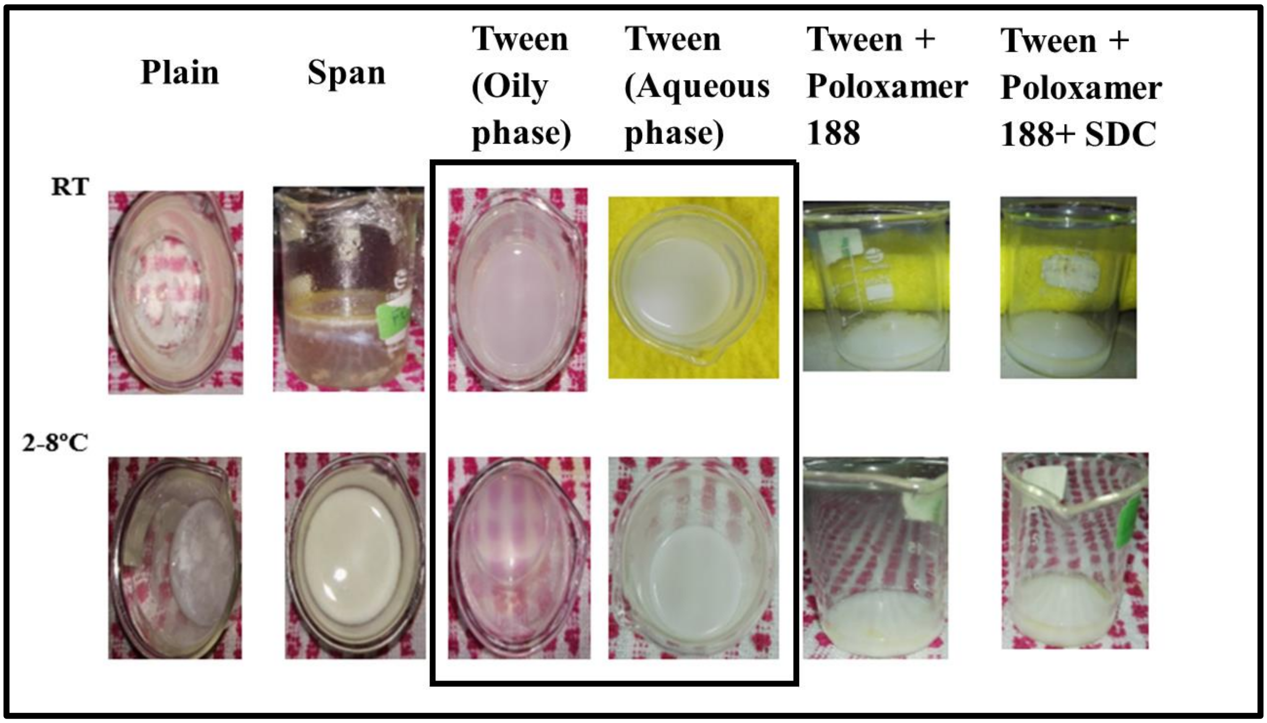

Figure S2: Images of the visual inspection of the influence of surfactant type on phosalosomal dispersion after storing at RT and at 2-8°C for two weeks.

Table S1: Particle size, PDI, and zeta-potential of freshly prepared ETD-phosalosomal dispersion (ETD-PHs) after 3 month stored formulation. Results expressed as an average of triplicate readings ± SD.

Time	Size (nm)	ζ potential(mV)	PDI
Fresh	277.44±6.98	-23.44±1.31	0.416±0.013
After 3 months	263.15±5.44	-21.85±1.41	0.470±0.021

A

B

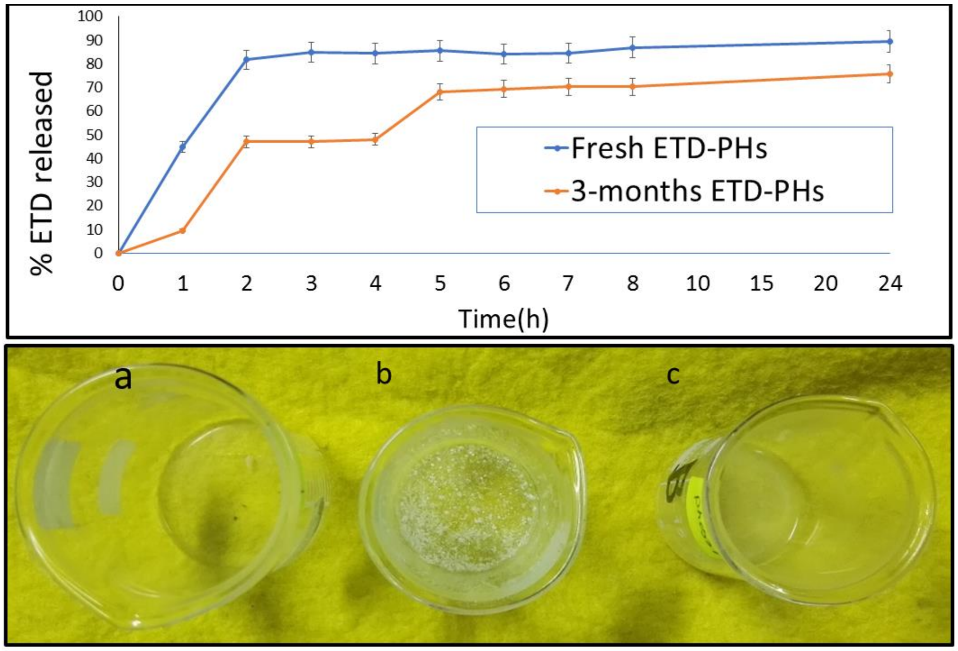

Figure S3: (A) In-vitro ETD release profiles from Freshly and 3-month stored Phosalosomal dispersion (ETD-PHs) in PB (pH5.5) at 32±0.5°C for 24 h using dialysis-bag method.(B) Stability of a) plain hyaluronic acid gel, b) ETD-HA gel , c)ETD-PHs-HA gel after 3 months.

References

1. Hafez DA, Abdelmonsif DA, Aly RG, Samy WM, Elkhodairy KA, Abo Aasy NK. Role of fennel oil/ quercetin dual nano-phytopharmaceuticals in hampering liver fibrosis: Comprehensive optimization and in vivo assessment. Journal of Drug Delivery Science and Technology. 2022;69.
2. Ridolfi DM, Marcato PD, Justo GZ, Cordi L, Machado D, Durán N. Chitosan-solid lipid nanoparticles as carriers for topical delivery of tretinoin. Colloids and Surfaces B: Biointerfaces. 2012;93:36-40.
3. Barakat NS. Etodolac-liquid-filled dispersion into hard gelatin capsules: an approach to improve dissolution and stability of etodolac formulation. Drug development and industrial pharmacy. 2006;32(7):865-76.
